# High Throughput Screen for *Escherichia coli* Twin Arginine Translocation (Tat) Inhibitors

**DOI:** 10.1371/journal.pone.0149659

**Published:** 2016-02-22

**Authors:** Umesh K. Bageshwar, Lynn VerPlank, Dwight Baker, Wen Dong, Shruthi Hamsanathan, Neal Whitaker, James C. Sacchettini, Siegfried M. Musser

**Affiliations:** 1 Department of Molecular and Cellular Medicine, College of Medicine, Texas A&M Health Science Center, College Station, TX, United States of America; 2 Broad Institute, Cambridge, MA, United States of America; 3 Department of Biochemistry and Biophysics, Texas A&M University, College Station, TX, United States of America; Centre National de la Recherche Scientifique, Aix-Marseille Université, FRANCE

## Abstract

The twin arginine translocation (Tat) pathway transports fully-folded and assembled proteins in bacteria, archaea and plant thylakoids. The Tat pathway contributes to the virulence of numerous bacterial pathogens that cause disease in humans, cattle and poultry. Thus, the Tat pathway has the potential to be a novel therapeutic target. Deciphering the Tat protein transport mechanism has been challenging since the active translocon only assembles transiently in the presence of substrate and a proton motive force. To identify inhibitors of Tat transport that could be used as biochemical tools and possibly as drug development leads, we developed a high throughput screen (HTS) to assay the effects of compounds in chemical libraries against protein export by the *Escherichia coli* Tat pathway. The primary screen is a live cell assay based on a fluorescent Tat substrate that becomes degraded in the cytoplasm when Tat transport is inhibited. Consequently, low fluorescence in the presence of a putative Tat inhibitor was scored as a hit. Two diverse chemical libraries were screened, yielding average Z'-factors of 0.74 and 0.44, and hit rates of ~0.5% and 0.04%, respectively. Hits were evaluated by a series of secondary screens. Electric field gradient (Δψ) measurements were particularly important since the bacterial Tat transport requires a Δψ. Seven low *IC*_*50*_ hits were eliminated by Δψ assays, suggesting ionophore activity. As Δψ collapse is generally toxic to animal cells and efficient membrane permeability is generally favored during the selection of library compounds, these results suggest that secondary screening of hits against electrochemical effects should be done early during hit validation. Though none of the short-listed compounds inhibited Tat transport directly, the screening and follow-up assays developed provide a roadmap to pursue Tat transport inhibitors.

## Introduction

The targeting and transport of proteins across lipid membrane barriers is a fundamental process in all cells that is essential for growth, development and homeostasis. In general, ~30–50% of an organism's proteome is transported across or inserted into membranes by a variety of protein translocation machineries [1,2]. In bacteria, most extra-cytoplasmic proteins are transported across or into the cytoplasmic membrane by one of two major pathways, the general secretory (Sec) pathway [3] or the twin arginine translocation (Tat) pathway [4].

The Tat pathway is unique because it transports fully-folded and assembled proteins (i.e., large, irregular-shaped macromolecules) without compromising the membrane's role as a barrier to ions and metabolites [4]. In addition, it requires the presence of a proton motive force (PMF) to function, but not nucleoside triphosphates (NTPs) [5,6]. In bacteria, only the electrical field gradient (Δψ) component of the PMF is required for Tat transport [7]. The N-terminal signal peptides (or ‘presequences’) of Tat precursor proteins contain a twin arginine consensus motif (RR-motif)–hence the name Tat, for twin-arginine translocation [8]. In the bacterial consensus motif, (S/T)RRxFLK, the arginine residues are almost invariant, whereas the other amino acid residues occur with a frequency of > 50%. About 8% of *E*. *coli* cell-envelope proteins (~30) are transported by the Tat machinery, and ~2/3 of these proteins contain prosthetic groups, which are inserted into the proteins in the cytoplasm [9,10].

The *E*. *coli* Tat protein transport system contains four identified protein components, TatA, TatB, TatC and TatE. Three of these proteins—TatA, TatB and TatE—are structurally similar, and likely have a common origin [11]. They each have a single N-terminal transmembrane domain and a C-terminal cytoplasmic domain [4,12]. TatC has six transmembrane domains with both N- and C-termini facing the cytoplasm [13–15]. TatBC oligomers form the receptor complex for Tat substrates [16,17]. The TatC X-ray structure reveals a glove-shaped pocket, which can potentially accommodate a signal peptide hairpin that partially spans the bilayer [15,18]. TatA and TatE form homo-oligomeric rings [19], suggesting that these proteins can form translocation channels. The dominant model hypothesizes that TatA (or TatE) is recruited to the TatBC-substrate complex in the presence of a PMF and forms the conduit necessary for cargo transport [19,20]. Small molecules that perturb, inhibit or stabilize intermediates in this process are expected to be quite useful for deciphering the transport mechanism and/or as *in vitro* or *in vivo* tools.

The Tat pathway is important for the pathogenicity of many bacteria [21]. In the case of *M*. *tuberculosis*, which is responsible for ~2 million global deaths annually due to tuberculosis, the Tat pathway appears essential for bacterial survival [22,23]. Though the Tat machinery is not essential for the growth of most bacteria, Tat deletion strains usually exhibit significant growth defects such as enhanced susceptibility to external agents (antibiotics and detergents), and/or reduced virulence [24–27]. The widespread increase in drug-resistant pathogens, especially many multidrug-resistant strains, and the shortage of new antimicrobials emphasizes the importance of discovering and characterizing new antibiotics and antibiotic targets [28]. In animals, the Tat system has only been found in aspiculate homoscleromorph sponges [29]. Since the Tat machinery is absent from higher animals including humans, a drug that inhibits protein export through the Tat machinery with high specificity is expected to be effective in neutralizing or attenuating the pathogenicity of numerous bacteria, possibly without, or with few, side effects.

We describe herein a high throughput screening approach to identify small molecule inhibitors of Tat transport that can be used to assist with the characterization and elucidation of the Tat transport mechanism and as lead compounds for the development of antibiotics to benefit human health. This screen is based on a fluorescent Tat substrate that becomes degraded when Tat transport is inhibited. We screened 51,600 compounds from a local library and 337,881 compounds from the NIH's Molecular Libraries Small Molecule Repository (MLSMR) at the Broad Institute Probe Development Center (BIPDeC) (Cambridge, MA), a member of the Molecular Libraries Probe Production Centers Network (MLPCN). The majority of lead compounds obtained from the primary assay did not survive confirmation and dose response, and the remaining compounds did not survive follow-up biochemical assays. Compared with a previous approach [30], the strengths of our screening protocol are *in vitro* biochemical assays that can verify whether the Tat machinery is a direct target of a putative inhibitor.

## Results

### Design of the HTS Assay

**Fig 1:**
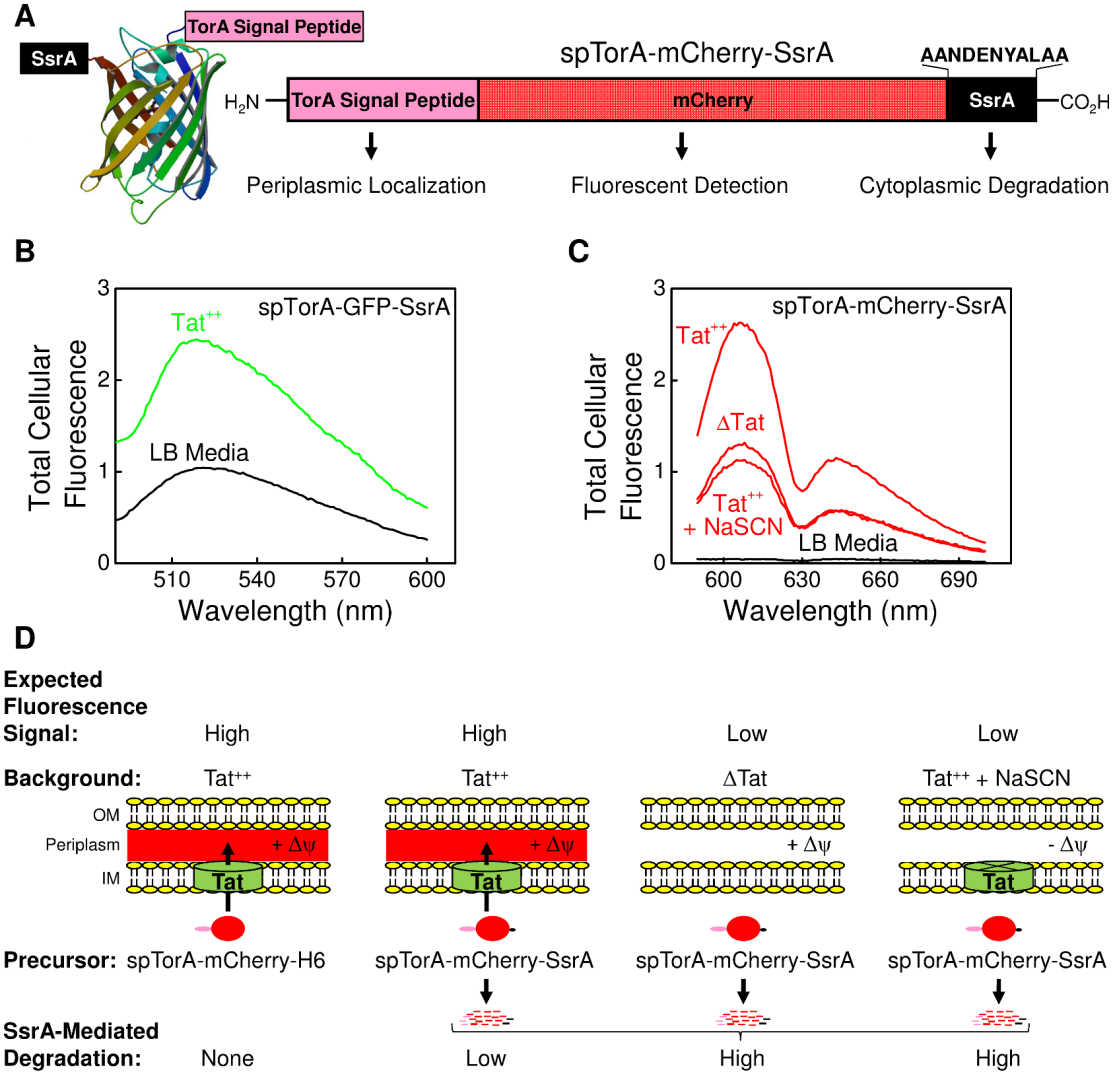
Design of the HTS. (A) The fluorescent Tat precursor protein spTorA-mCherry-SsrA. The N-terminal TorA signal peptide (spTorA) targets the fluorescent protein mCherry (PDB: 2H5W) to the Tat machinery for transport to the periplasm, and the C-terminal SsrA-tag promotes cytoplasmic degradation of the protein. (B) Fluorescence spectra of LB media alone (*black*) and with cells producing spTorA-GFP-SsrA in the Tat^++^ background (*green*) (EX = 485 nm). Tat^++^ denotes strain MC4100(DE3) in which TatABC is overproduced from the pTatABC-Duet1 plasmid (see Methods). (C) Fluorescence spectra of LB media alone (*black*) and with cells producing spTorA-mCherry-SsrA (*red*) under the indicated conditions (EX = 587 nm). NaSCN collapses the Δψ across the inner membrane. For (B) and (C), cells with (Tat^++^) or without (ΔTat) TatABC were induced with the indicated proteins and then incubated for 12 h at 4°C (ΔTat denotes strain MC4100ΔTatABCDE; see Methods for more details). (D) Design of the HTS assay. The SsrA tag on spTorA-mCherry-SsrA promotes degradation of cytoplasmically-localized spTorA-mCherry-SsrA, and thus, periplasmically-localized mature Tat cargo (mCherry-SsrA) is the dominant contributor to the total mCherry cellular fluorescence. In the ΔTat background, or in the presence of 30 mM NaSCN (positive control; simulated hit), the total cellular fluorescence is lower, as shown in (C). Due to the competition between transport of mCherry to the periplasm by the Tat machinery and degradation by the ClpXP/ClpAP protease system, low fluorescence in the presence of a putative Tat inhibitor was considered a hit. spTorA-mCherry-H6 (replacing the SsrA tag with a 6xHis-tag) is a control protein that is not degraded in the cytoplasm. OM = outer membrane; IM = inner membrane.

We developed a live cell-based high throughput screen (HTS) in which a C-terminal SsrA tag promotes the cytoplasmic degradation of a pre-protein when Tat-dependent protein export is inhibited or blocked. It was previously demonstrated that when Tat-dependent export of spTorA-GFP-SsrA (which consists of the signal peptide of TorA fused to GFP with a C-terminal SsrA tag) is impaired, the protein remaining in the cytoplasm is degraded by the ClpXP/ClpAP protease system [31]. Since the fluorescence emission of GFP overlaps significantly with the intrinsic fluorescence of Luria-Bertani (LB) media, we replaced the GFP domain with mCherry, designating the new fluorescent Tat substrate as spTorA-mCherry-SsrA (Fig 1A and 1B, S1 Fig). The fluorescence emission from mCherry does not overlap with the intrinsic fluorescence of LB media (Fig 1C).

The N-terminal signal peptide of TorA (spTorA) targets spTorA-mCherry-SsrA to the Tat system for export to the periplasm. At the same time, the C-terminal SsrA tag promotes ClpXP/ClpAP-dependent degradation of spTorA-mCherry-SsrA protein molecules in the cytoplasm. Thus, there is competition between the export and degradation pathways. The total cellular fluorescence arises from cytoplasmically-localized (and not degraded) mCherry protein (predominantly the full-length precursor spTorA-mCherry-SsrA) and periplasmically-localized mature protein (mCherry-SsrA). Due to the degradation of cytoplasmically-localized spTorA-mCherry-SsrA, the total mCherry fluorescence observed for wild type cell cultures should arise primarily from periplasmically-localized mCherry-SsrA. In contrast, when the Tat machinery is absent or blocked (e.g., by an inhibitor), a significant decrease in fluorescence is expected (Fig 1D).

### Optimization of the Total mCherry Fluorescence Signal and Validation of Substrate Design Properties

**Fig 2:**
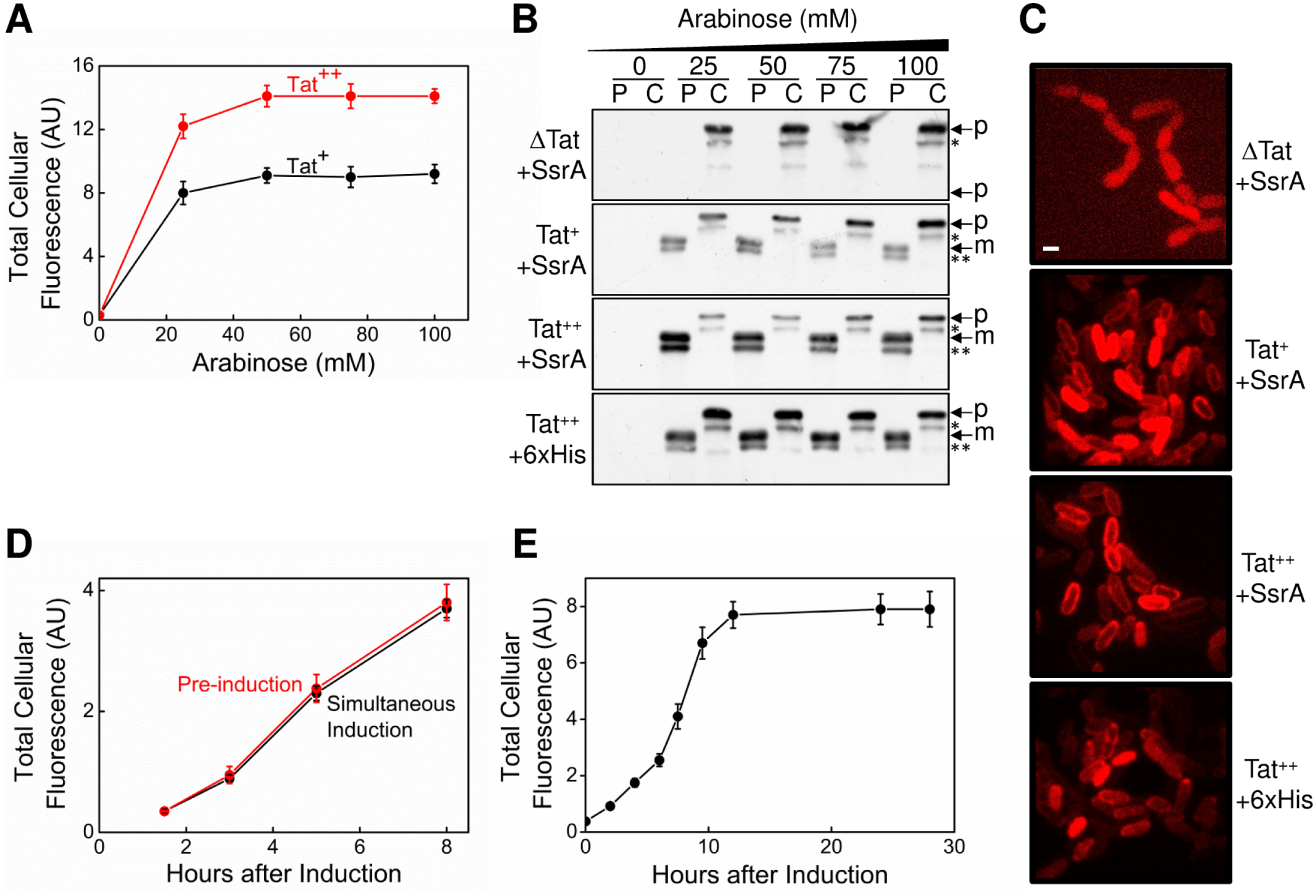
Optimization of the Total mCherry Fluorescence Signal and Validation of Substrate Design Properties. (A) Effect of spTorA-mCherry-SsrA induction levels on the total mCherry cellular fluorescence in wild type (Tat^+^; MC4100(DE3)) and TatABC overproduced (Tat^++^) backgrounds. The production of spTorA-mCherry-SsrA was induced for 8 h at 25°C with the indicated concentrations of arabinose and the total mCherry cellular fluorescence was determined (*n* = 3; EX = 587 nm, EM = 610 nm). TatABC was induced with 1 mM IPTG. (B) Cell fractionation. Cytoplasmic (C) and periplasmic (P) fractions [60] of the indicated strains were analyzed after 8 h induction by SDS-PAGE and immunoblotting using anti-mCherry antibodies. For the top three gels, the cells overproduced spTorA-mCherry-SsrA. For the bottom gel, the SsrA tag on spTorA-mCherry-SsrA was replaced with a 6xHis-tag (yielding spTorA-mCherry-H6). Precursor (p) and mature (m) proteins are indicated. The * and ** identify what appears to be C-terminally truncated products. (C) Fluorescence microscopy of the strains in (B). The mCherry proteins were induced at 25°C for 15 h with 2 mM arabinose. Cells were grown in fresh media for an additional 5 h with no arabinose before imaging. More than 95% of cells showed a clear and dominating periplasmic localization of mCherry in the Tat^++^ strain versus ~70% for the Tat^+^ strain. No periplasmic mCherry was observed in the ΔTat strain, confirming that the spTorA-mCherry-SsrA protein was indeed targeted to and transported by the Tat system. Replacing the SsrA tag with a 6xHis-tag resulted in an increase in the amount of cytoplasmic mCherry (compare with Fig 2B), confirming that the SsrA tag promotes cytoplasmic degradation of the spTorA-mCherry-SsrA protein. Visually, about 80% of cells showed noticeable cytoplasmic localization of spTorA-mCherry-H6. Bar = 1.3 μm. (D) Effect of TatABC pre-induction on the total cellular fluorescence of mCherry. TatABC was induced 3 h prior to or simultaneously with spTorA-mCherry-SsrA (100 mM arabinose; *n* = 3). (E) Effect of induction time on the total cellular fluorescence of mCherry. The spTorA-mCherry-SsrA protein was produced in the Tat^++^ background and the total mCherry cellular fluorescence was obtained as in (A) (*n* = 3).

For maximum sensitivity in a HTS, a strong signal is needed. We examined the strength of the mCherry fluorescence signal when the spTorA-mCherry-SsrA induction level was varied (0–100 mM arabinose) in wild type (Tat^+^) and TatABC overproduced (Tat^++^) backgrounds. The total cellular fluorescence plateaued at 50–100 mM arabinose, and TatABC overproduction yielded an ~50% greater signal (Fig 2A). Cell fractionation and fluorescence microscopy analysis confirmed that the major fluorescent species in the Tat^++^ (spTorA-mCherry-SsrA) strain was periplasmically-localized mature protein (Fig 2B and 2C). Replacing the SsrA tag with a 6xHis-tag (yielding spTorA-mCherry-H6) resulted in an increase in cytoplasmic mCherry, confirming that the SsrA tag promotes cytoplasmic degradation of the spTorA-mCherry-SsrA protein. No periplasmic mCherry was observed in a Tat-deficient strain (ΔTat), confirming that the spTorA-mCherry-SsrA protein was indeed targeted to and transported by the Tat system (Fig 2B and 2C). No difference in total cellular fluorescence was observed if TatABC overproduction was induced at the same time as or 3 h before spTorA-mCherry-SsrA induction (Fig 2D). The total cellular fluorescence plateaued ~12 h after spTorA-mCherry-SsrA production was induced (Fig 2E). In summary, these data validate the spTorA-mCherry-SsrA protein as a suitable fluorescence substrate for use in a HTS.

### Stability of the mCherry Fluorescence Signal

**Fig 3:**
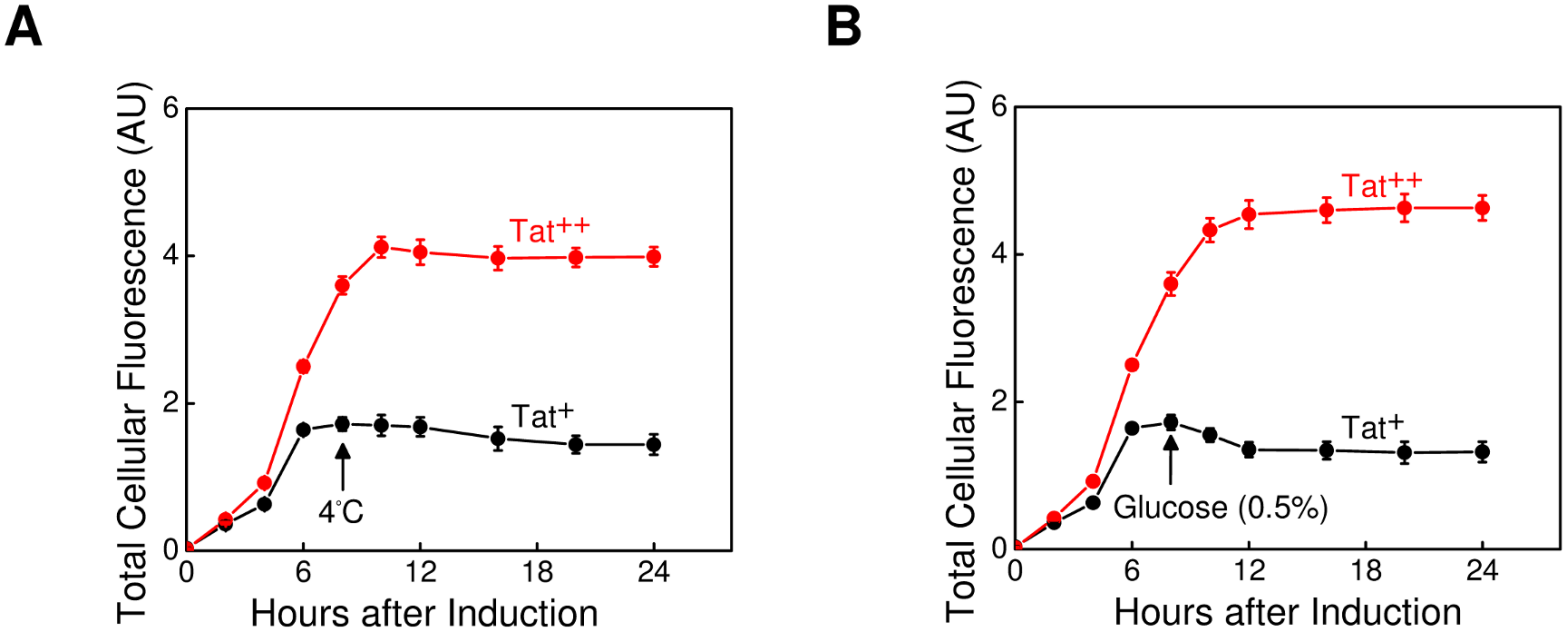
Stability of the mCherry Fluorescence Signal. (A) Stability of the total cellular fluorescence after transfer to 4°C. Cells producing spTorA-mCherry-SsrA under Tat^+^ or Tat^++^ conditions were incubated at 25°C for 8 h, and then transferred to 4°C to inhibit growth. The total mCherry cellular fluorescence was monitored periodically (*n* = 3). (B) Stability of the total cellular fluorescence after glucose addition. The total cellular fluorescence was quantified as in (A), except that spTorA-mCherry-SsrA production was repressed by addition of 0.5% glucose after 8 h of growth. Growth was continued at 25°C (*n* = 3). These data demonstrate that the intensity of the mCherry fluorescence signal can be maintained at a constant value for long time periods, thereby providing consistency when reading many HTS plates.

Since large HTSs require lengthy read times, we examined the stability of the mCherry fluorescence signal under Tat^+^ and Tat^++^ conditions. After 8 h of induction, spTorA-mCherry-SsrA production was repressed by incubating cultures at 4°C (Fig 3A), which blocks cell growth, or by the addition of 0.5% glucose (Fig 3B), which represses the arabinose promoter [32]. Both lower temperature and glucose addition stabilized the total cellular fluorescence signal for at least ~12 h, although there were lags of ~2 and ~4 h before the fluorescence signal stabilized under low temperature and glucose repression conditions, respectively. These experiments identified a timeframe in which we could read plates with minimal intra-plate and plate-to-plate variation.

### Effect of Oxidative Phosphorylation Inhibitors on *In Vivo* Tat Transport

**Fig 4:**
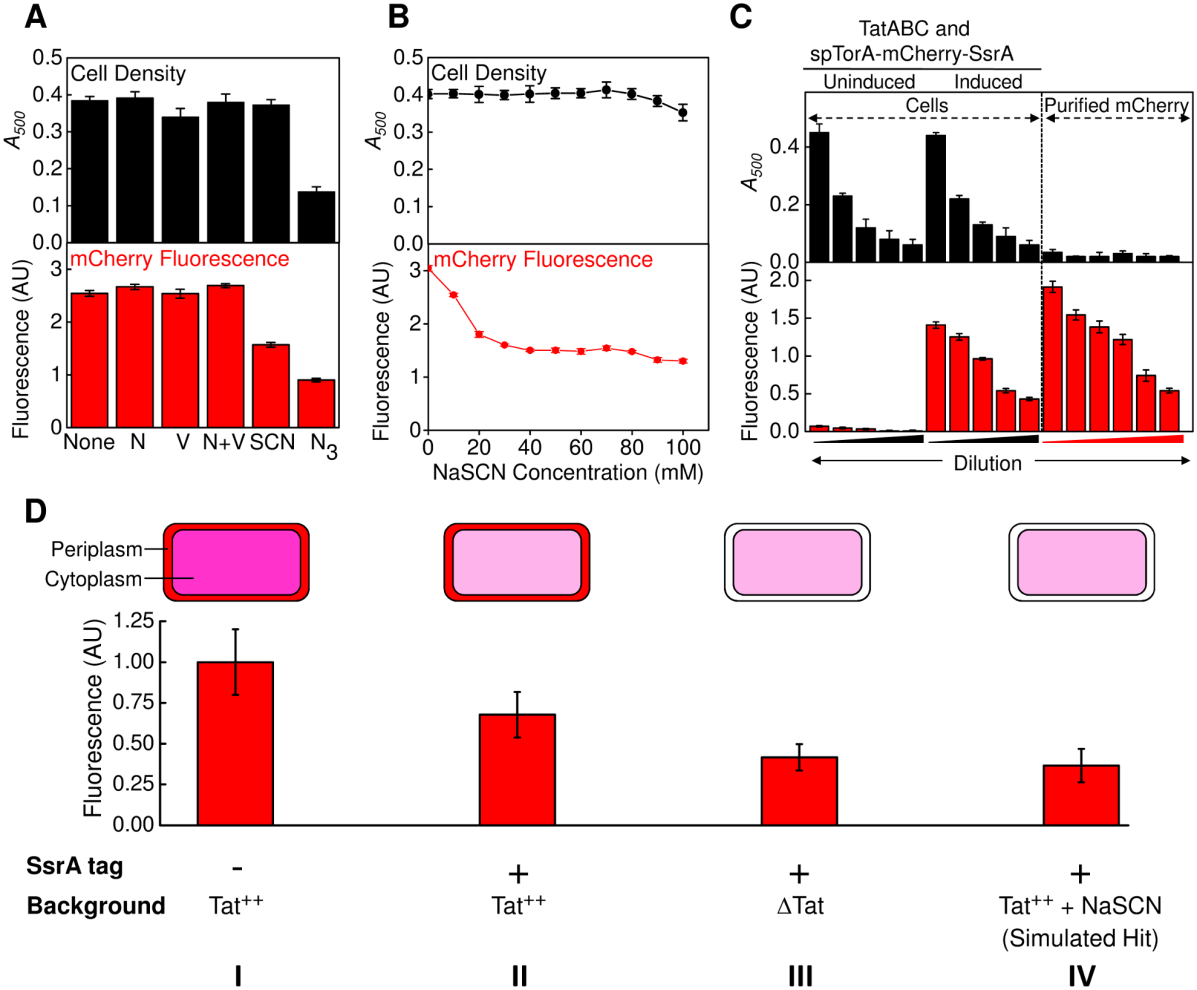
Effect of Oxidative Phosphorylation Inhibitors on *In Vivo* Tat Transport and Sensitivity of Fluorescence Detection on Plates. (A) Effect of oxidative phosphorylation inhibitors on cell growth and mCherry fluorescence under Tat^++^ conditions. Cells producing spTorA-mCherry-SsrA under Tat^++^ conditions were grown at 25°C for 8 h in the presence of the indicated respiratory inhibitors. After overnight incubation at 4°C, optical densities (*upper panel; A*_*500*_) and total mCherry cellular fluorescence intensities (*lower panel*; EX = 587 nm, EM = 610 nm) were measured (*n* = 3). Nigericin (N; 50 μM) is a H^+^/K^+^ exchanger, which collapses the ΔpH gradient. Valinomycin (V; 50 μM) is a K^+^ ionophore, which collapses the Δψ. NaSCN (SCN; 50 mM) collapses the Δψ. NaN_3_ (N_3_; 0.1%) inhibits the cytochrome *bo*_*3*_ terminal oxidase. (B) Effect of NaSCN concentration on cell density and total mCherry cellular fluorescence. Measurements were made after incubating cells overnight at 4°C (*n* = 3). (C) Calibration and sensitivity of mCherry fluorescence detection in 384-well plates. Overnight cultures of *E*. *coli* MC4100(DE3) cells were diluted to *A*_*500*_ = 0.25, 0.2, 0.15, 0.10 and 0.05, and aliquoted into 384-well plates. The spTorA-mCherry-SsrA and TatABC proteins were induced together or neither were induced, as indicated. The cells were incubated at 25°C for 8 hours, and stored overnight at 4°C. Optical densities (*upper panel*) and mCherry fluorescence intensities (*lower panel*) were determined with a BMG Polarstar Omega plate reader (at 500 nm for absorbance; EX = 584 nm, EM = 620 nm for fluorescence). For comparison, the absorbance and fluorescence intensities from purified spTorA-mCherry-H6 (30, 25, 20, 15, 10 and 5 nM) are also shown (*n* = 3 plates, 16 wells/plate). (D) Simulated hits on 384-well plates. Using the approach described in (C), cells (*A*_*500*_ = 0.15) were grown on plates under the indicated conditions, and then fluorescence intensities were measured. spTorA-mCherry-SsrA was used for conditions II-IV. spTorA-mCherry-H6 was used for a minus SsrA control (I; 100%). The SsrA tag decreased the mCherry fluorescence intensity (II; 63±14%), consistent with a decrease in cytoplasmic mCherry concentration due to SsrA-dependent degradation. A further decrease in mCherry fluorescence intensity was observed in Tat-deficient cells (ΔTat) (III; 41±6%) and in the presence of 30 mM NaSCN (IV; 36±14%), consistent with enhanced cytoplasmic degradation due to inhibition of export to the periplasm (*n* = 3 plates, 16 wells/plate).

When screening for inhibitors via HTS, it is useful to include a known inhibitor as a positive control. Since the Δψ is required for bacterial Tat transport [7], we tested whether various respiration inhibitors could be used to collapse the Δψ *in vivo*. *In vitro*, Tat transport is completely inhibited by the addition of both nigericin and valinomycin, which collapse the transmembrane pH gradient (ΔpH) and the Δψ, respectively [7]. High concentrations of nigericin and/or valinomycin (50 μM each) did not affect cell growth or mCherry fluorescence in culture (Fig 4A). This was unexpected since 2.5 μM of each these two ionophores are sufficient to completely block Tat transport *in vitro* [7]. NaSCN alone completely inhibits *in vitro* Tat transport, and, like valinomycin, NaSCN collapses the Δψ [7]. NaSCN (50 mM) reduced mCherry fluorescence by ~40% without affecting growth of *E*. *coli* cultures (Fig 4A). Dose response data showed that 30 mM NaSCN is sufficient to maximally reduce the mCherry fluorescence signal without affecting cell growth (Fig 4B). Thus, NaSCN appears more effective than valinomycin at inhibiting the Δψ *in vivo*, resulting in reduced Tat-dependent protein transport. Consequently, NaSCN was used throughout our screens as a positive control (simulated hit). Sodium azide is an inhibitor of cytochrome *bo*_*3*_, the terminal oxidase in the aerobic respiratory chain of *E*. *coli* [33–35]. As expected, azide inhibited cell growth and decreased the mCherry fluorescence signal by similar amounts (Fig 4A).

### Sensitivity of Fluorescence Detection on Plates

We next tested whether cellular mCherry fluorescence could be detected at reasonable cell densities after growth in 384-well plates. Tat^++^ cells producing spTorA-mCherry-SsrA were serially diluted into 384-well plates and incubated at 25°C for 8 h. Dose-dependent absorbance (due to light scatter) and fluorescence signals were obtained. Under uninduced conditions, the fluorescence signals were negligible, but cell densities were unaffected, as expected. Purified spTorA-mCherry yielded a dose-dependent fluorescence signal and negligible *A*_*500*_, also as expected (Fig 4C). These results demonstrated a detectable range of cellular mCherry fluorescence signals on plates.

### Outline of the HTS Assay

Having obtained the calibration and validation data described in the previous sections, we next established the protocol for the HTS. Details are provided in the Materials and Methods section. Here, we briefly summarize the assay. A single colony of *E*. *coli* strain MC4100(DE3) containing plasmids encoding TatABC and spTorA-mCherry-SsrA was inoculated into LB and grown overnight. The next morning, cells were transferred to fresh media, the production of TatABC and spTorA-mCherry-SsrA was induced, and cultures were aliquoted onto plates with test compounds. NaSCN was included in control wells to simulate a hit. Cells were grown for 8 h, followed by incubation at 4°C overnight, and then read the next day. Setting the total cellular fluorescence obtained with spTorA-mCherry-H6 under Tat^++^ conditions to 100%, spTorA-mCherry-SsrA yielded a total cellular fluorescence signal of 63±14%, consistent with SsrA-induced degradation of the mCherry protein. The total cellular fluorescence was further reduced to 36±14% in the presence of 30 mM NaSCN (simulated hit), and to 41±6% in the absence of TatABC, verifying the Δψ- and Tat-dependence of the fluorescence signal (Fig 4D).

### The Local HTS

**Fig 5:**
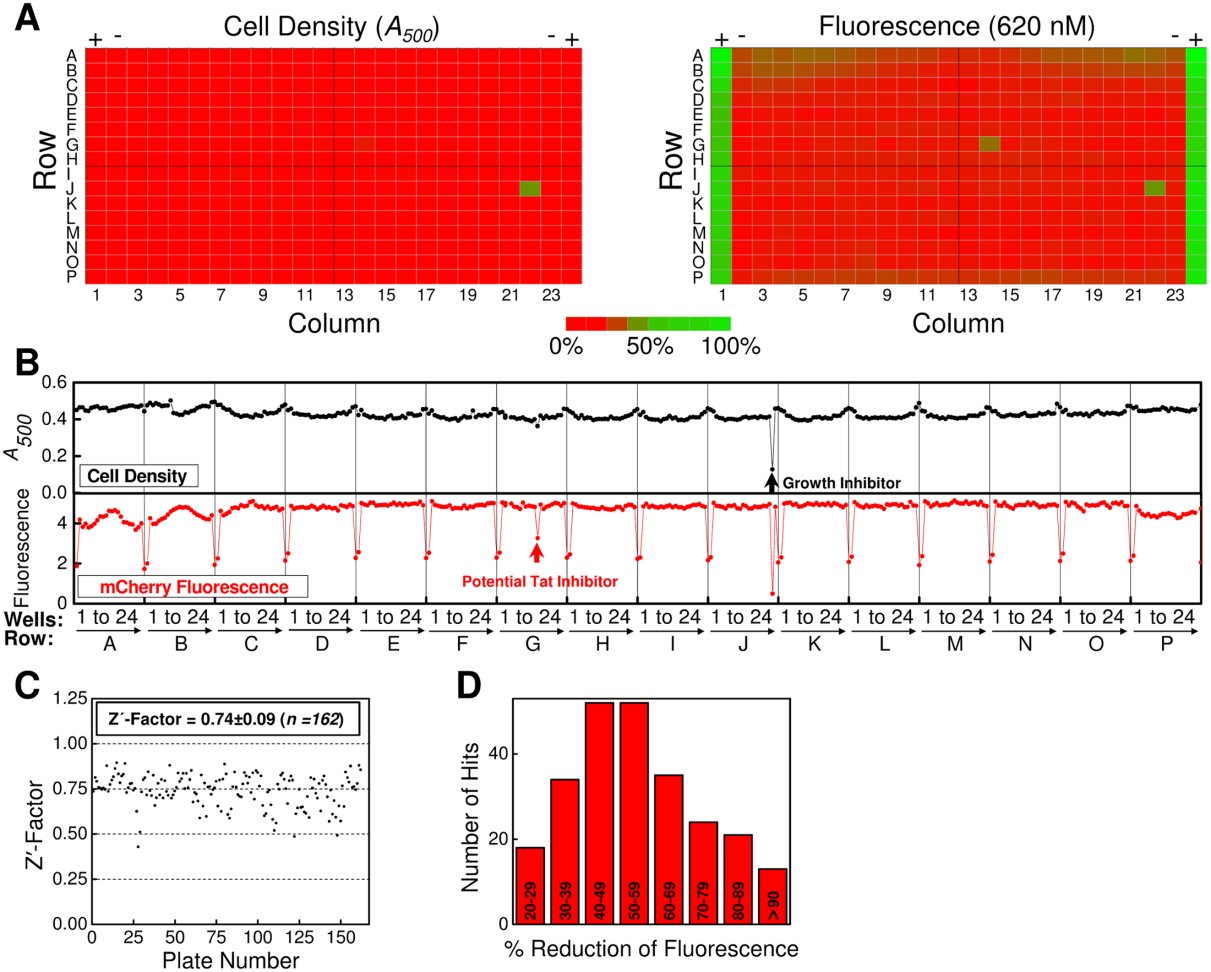
Sample Data from the Local HTS, Z'-factor Summary and Hit Summary. (A) Optical density (*left panel*; *A*_*500*_) and fluorescence intensity (*right panel*; EX = 584 nm; EM = 620 nm) values from a typical local HTS plate obtained under Tat^++^ conditions. DMSO only (*columns 2* and *23*) and 30 mM NaSCN (*columns 1* and *24*) controls are identified as − and +, respectively. (B) Graphical representation of the optical density (*black*) and fluorescence intensity (*red*) values for each well of the plate shown in (A). A potential Tat inhibitor (*well G-14*) and growth inhibitor (*well J-22*) are identified by *red* and *black* arrows, respectively. These intensity signatures are detectable in the raw data images in (A). (C) The Z'-factor for each plate from the local HTS. (D) Fluorescence intensity distribution of compounds with the intensity signatures expected for a Tat inhibitor (see text). The percent fluorescence decrease is normalized to the 30 mM NaSCN positive control (100%) and the DMSO control (0%).

Preliminary HTS testing and optimization, initial small test screens, and a HTS of 51,600 diverse synthetic compounds were performed in the laboratory of one of us (James Sacchettini). A sample 384-well plate from this local HTS is shown in Fig 5A. The edge columns contain DMSO (−; negative control) and 30 mM NaSCN (+; positive control). Compounds were tested at 5 or 20 μM. In this HTS, all plates were scanned for both cell growth (*A*_*500*_) and mCherry fluorescence (Fig 5A and 5B). The control wells on each plate were used to calculate the Z'-factor [36] for each plate, which provides an assessment of the performance of the assay on each plate. The average Z'-factor was 0.74±0.09 (Fig 5C; *n* = 162 plates), indicating a robust HTS assay. Some edge effects were observed (e.g., see Fig 5B), likely from uneven aeration or heating across the plate during the growth incubation period, but these did not interfere with hit calling.

Three categories of non-baseline outputs were identified. The first was *“low cell density*”, the expected signature for growth inhibitors (77 compounds, 0.15% of total). The second category was “*high fluorescence and unchanged cell density”*, which was expected for fluorescent compounds, compounds that enhanced Tat translocation, compounds that increased transcription of the spTorA-mCherry-SsrA mRNA, and compounds that inhibited the ClpXP/ClpAP protease system that recognizes the SsrA tag (208 compounds, 0.41% of total). The third category was *“low fluorescence and unchanged cell density”*, the expected signature for true Tat inhibitors. Compounds exhibiting this signature were considered “hits” (249 compounds, 0.48% of total).

Hits were defined as having a ≥ 20% decrease in mCherry fluorescence, compared with the NaSCN control (100%). The first 12,800 compounds (plates 1–40) were screened using 5 μM of each test compound, yielding a hit rate of 0.016%. This was considered a low hit rate, so the remaining compounds in the library (38,800 compounds, plates 41–162) were assayed at 20 μM, increasing the hit rate to 0.63%. The median fluorescence decrease for hits was 65% (Fig 5D). The 249 hits were re-assayed via dose response (Fig 6), which yielded 78 surviving compounds.

**Fig 6:**
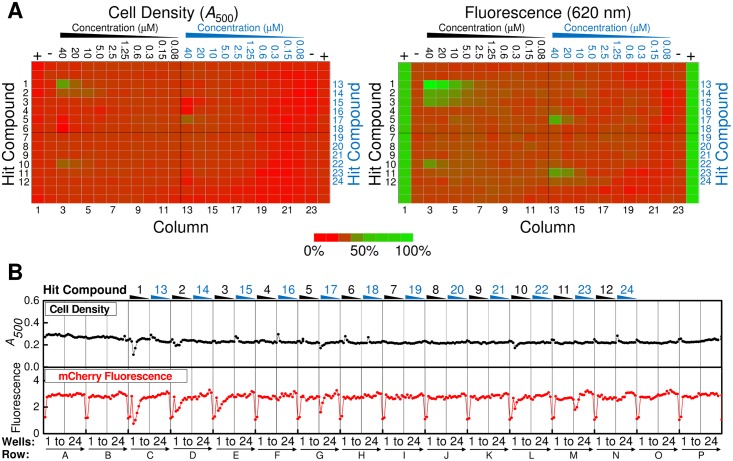
Sample Dose Response Data for Hits from the Local HTS. (A) Optical density (*left panel*) and fluorescence intensity (*right panel*) values for a typical local HTS dose response plate obtained under Tat^++^ conditions. DMSO only (*columns 2 and 23*) and 30 mM NaSCN (*columns 1 and 24*) controls are identified as − and +, respectively. Compound concentrations are given at the top of each column. Note that there are two different compounds per row. Dose response plates were run in duplicate. (B) Graphical representation of the values for the plate shown in (A).

### The Broad Institute Probe Development Center (BIPDeC) HTS

The validated 384-well HTS assay was transferred from the Texas A&M Health Science Center to the BIPDeC in order to screen the NIH’s Molecular Library Small Molecular Repository (MLSMR) collection of compounds. Approximately 20% of the compounds in the local chemical library were in common with the MLSMR collection at the time of screening. The HTS assay was adapted to run on the Broad’s high throughput screening system, which required further miniaturization to run in a 1536-well format. To make the primary assay run more efficiently, only the mCherry fluorescence was read, compared to the dual read done for the 384-well assay. From assay development and adaptation experiments, it was determined that glucose addition after the 8 h induction period followed by an overnight incubation at 4°C yielded a better signal-to-background ratio. The assay was validated using the MLSMR validation set, a small, ~2,000 compound collection composed of known bioactive compounds. The validation run showed strong statistics with an average signal-to-background ratio of 12.9, and an average Z'-factor [36] of 0.86 (data not shown).

**Fig 7:**
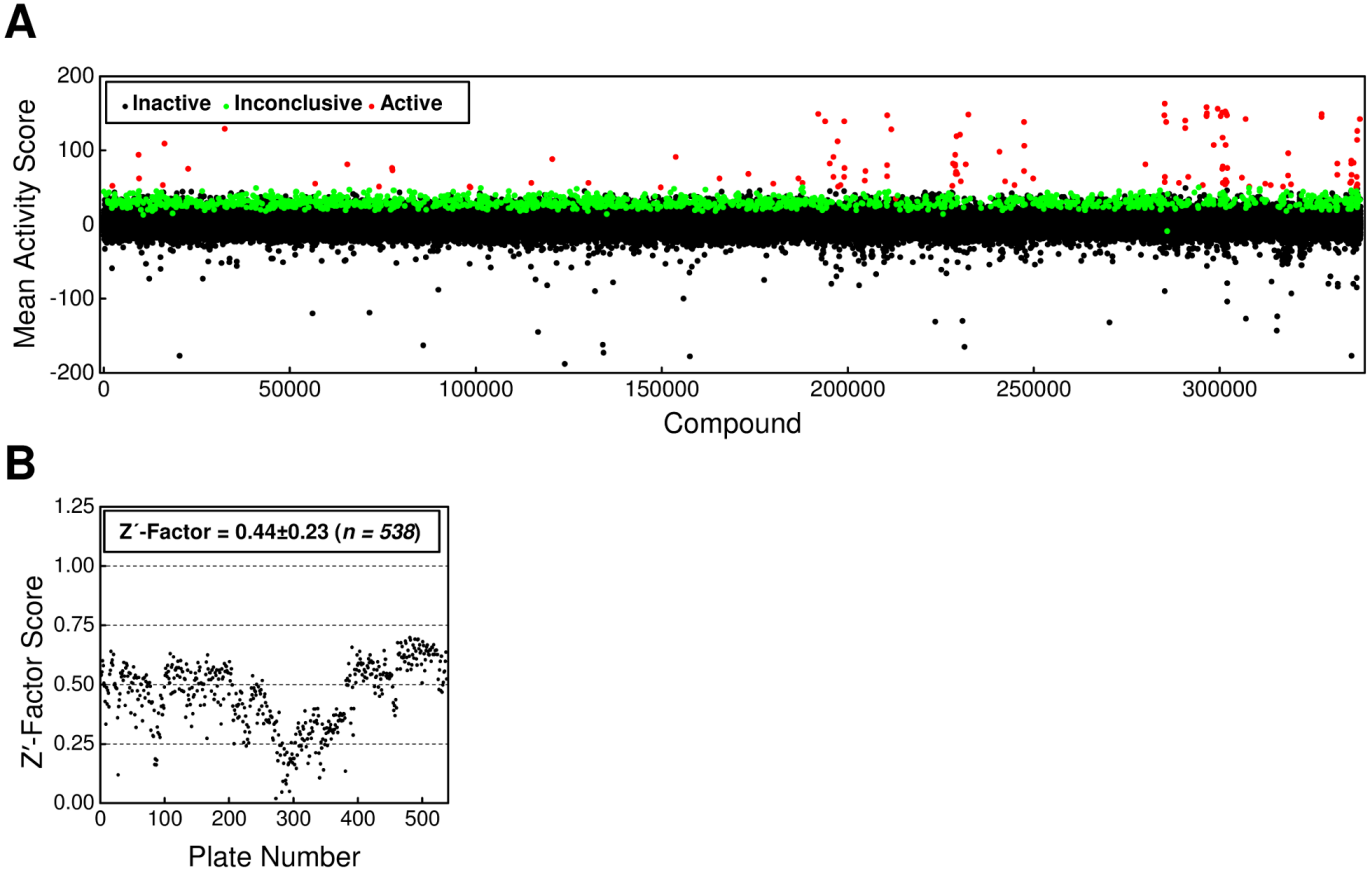
The BIPDeC HTS and Z'-factor Summary. (A) Activity results for the 337,881 compounds screened in the BIPDeC HTS. Active, inconclusive, and inactive activity scores are defined in the text. (B) The Z'-factor for each plate from the BIPDeC HTS.

Following validation, 337,881 compounds from the MLSMR collection were screened at 10 μM in duplicate using the adapted primary assay (Fig 7A). Tat translocation yield was assessed by the intensity of mCherry fluorescence. Raw fluorescence values were normalized to the average negative (DMSO only) and positive (30 mM NaSCN) controls on each plate. The average Z'-factor was 0.44±0.23 (Fig 7B; *n* = 538 plates). A mean activity score was calculated by averaging the fluorescence decrease observed for the two replicates of each test compound. Active compounds were defined as those compounds with a mean activity score ≥ 50 (50% reduction in fluorescence, normalized to the NaSCN and DMSO controls). According to this definition, 123 compounds were active (0.04% of the total screened). An additional 1,263 compounds were labeled as inconclusive, defined as those for which 1 of the 2 replicates had an activity score ≥ 50 (PubChem BioAssay AID 488895).

The active and inconclusive compounds were then reviewed for chemical intractability, and any compounds determined to be unsuitable for follow-up chemistry were removed from consideration. From this analysis, 1,256 compounds were reordered from the MLSMR for retesting with the primary assay at various concentrations. Of these compounds, 48 yielded an *IC*_*50*_ < 20 μM (PubChem BioAssays AID 504941 and 651752). The 1,256 compounds were also tested in a counter assay to identify non-specific bacterial inhibitors, which would show false positive responses in the primary assay. Bacterial viability was assayed using the Bac-TiterGlo (Promega) reagent using the same conditions as the primary assay, except without the addition of induction reagents. From this analysis, 309 compounds exhibited > 20% inhibition of bacterial growth (PubChem BioAssays AID 504843 and 651750). Based on the retest, counter-assay and a chemical suitability analysis, 35 potential inhibitors of Tat translocation were identified. These compounds were sourced from dry powders for additional retesting and secondary analysis.

### Secondary Screening: *In Vitro* Tat Transport Assays

Hits from the primary screen could include false positives for a variety of reasons. Hit compound effects on the spTorA-mCherry-SsrA reporter that would result in a reduced mCherry fluorescence signal include: i) a folding deficiency (i.e., which does not allow maturation of the mCherry fluorophore); ii) inhibition of transcription or translation; and iii) increased SsrA-dependent degradation (i.e., degradation instead of transport). In addition, Tat-dependent transport requires a Δψ [7]. Therefore, compounds that inhibit PMF generation would be indirect inhibitors of Tat-dependent transport. In contrast, our goal was to identify molecules that inhibited Tat transport by directly binding to Tat proteins. Additional screens were therefore required to verify whether hit compounds interacted specifically with Tat proteins.

To directly test for inhibition of Tat transport, a gel-based *in vitro* Tat transport assay was performed using pre-SufI, an authentic Tat precursor protein [7]. In short, this assay consists of incubating a Tat substrate and a Δψ-generating energy source with inverted membrane vesicles (IMVs) containing overproduced TatABC, digesting remaining external protein with a protease, and then analyzing via SDS-PAGE. This assay was run twice using NADH and ATP (if warranted) as energy sources to generate the necessary Δψ. NADH generates a Δψ using respiratory proteins (i.e., the NADH dehydrogenase and cytochrome *bo*_*3*_ ubiquinol oxidase complexes [35]) and ATP generates a Δψ by reversal of ATP synthase [37]. A true Tat inhibitor would survive both assays. In contrast, a respiration inhibitor is expected to reduce Tat transport when only one of the two energy sources is used (except for an ionophore).

**Fig 8:**
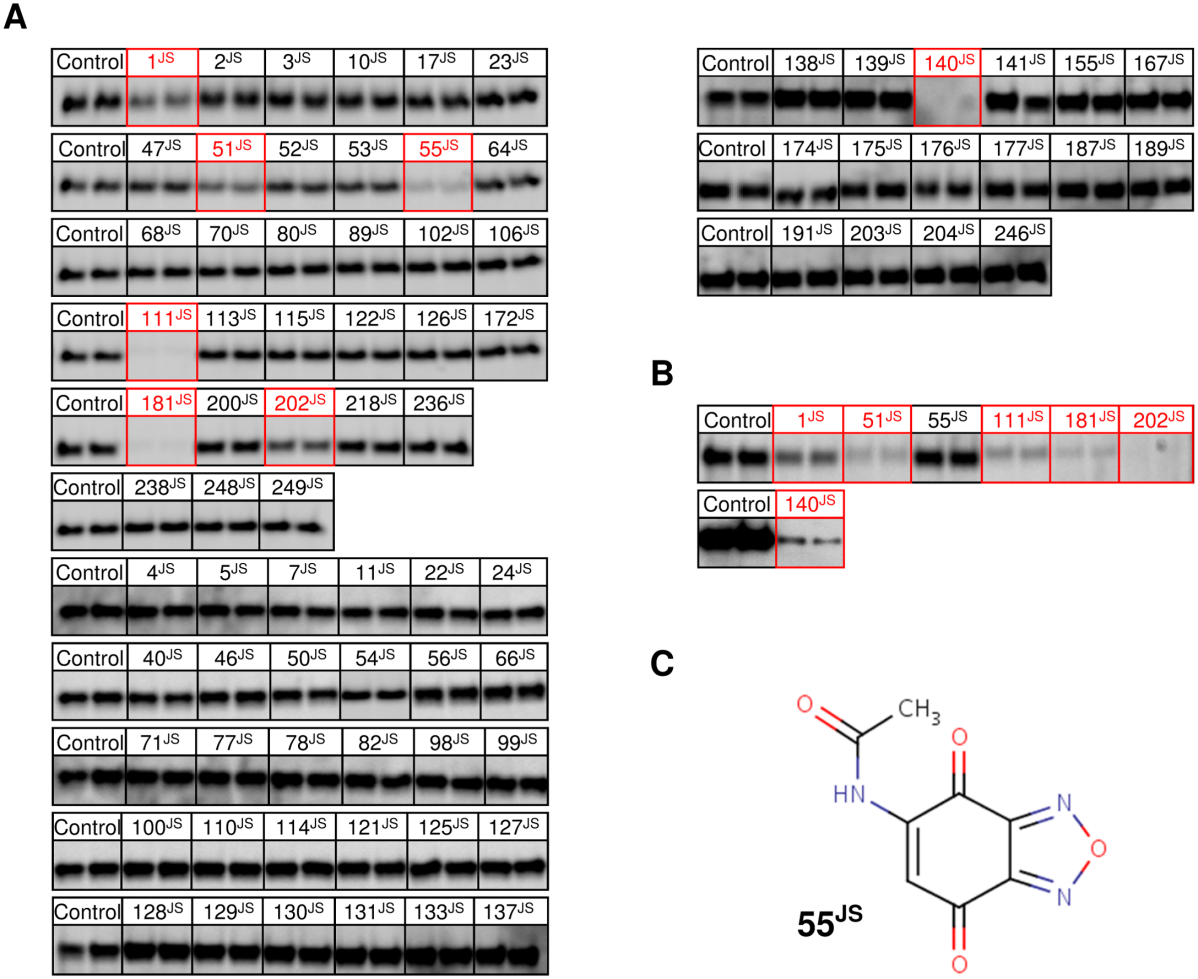
Effect of Hits from the Local HTS on *In Vitro* Tat Transport. (A) Tat transport assays of pre-SufI using NADH to generate a Δψ. Inverted membrane vesicles (IMVs; *A*_*280*_ = 5.0), the fluorescent substrate pre-SufI-IAC^Atto565^ (90 nM), and NADH (4 mM) were incubated at 37°C for 30 min with DMSO (control) or with hit compound (20 μM). Samples were treated with the protease proteinase K, run on SDS-PAGE, and analyzed by in-gel fluorescence imaging. Assays were run in duplicate. Band intensity corresponds to the amount of protein transported into the IMV lumen. The following 7 compounds survived this screen: 1^JS^, 51^JS^, 55^JS^, 111^JS^, 181^JS^, 202^JS^, and 140^JS^ (boxed in *red*). (B) Tat transport assays of pre-SufI using ATP to generate a Δψ. All 7 compounds surviving the screen in (A) were tested using a Tat transport assay performed identically to those in (A) except that ATP (1 mM) was used in place of NADH. Compound 55^JS^ was eliminated by this assay—the other 6 compounds survived (boxed in *red*). (C) Chemical structure of compound 55^JS^, N-(4,7-Dioxo-4,7-dihydro-2,1,3-benzoxadiazol-5-yl)acetamide. The quinone structure suggests NADH dehydrogenase as a possible target, leading to inhibition of Tat transport when using NADH as the energy source (A), but not when ATP is used (B).

The criterion for surviving the *in vitro* Tat transport assays was visible inhibition of Tat transport. Of the 78 compounds surviving the local HTS assay, 7 compounds (S2A–S2G Fig) survived the *in vitro* Tat transport assay with NADH (Fig 8A). Of these 7 compounds, 6 survived the ATP-dependent Tat transport assay (Fig 8B). Based on its elimination by the ATP-dependent assay, compound 55^JS^ (N-(4,7-Dioxo-4,7-dihydro-2,1,3-benzoxadiazol-5-yl)acetamide) appears to affect some step of aerobic respiration between NADH and O_2_. Since this compound is a quinone (Fig 8C), a likely possibility is that it interacts with the quinone binding site on NADH dehydrogenase [38,39].

**Fig 9:**
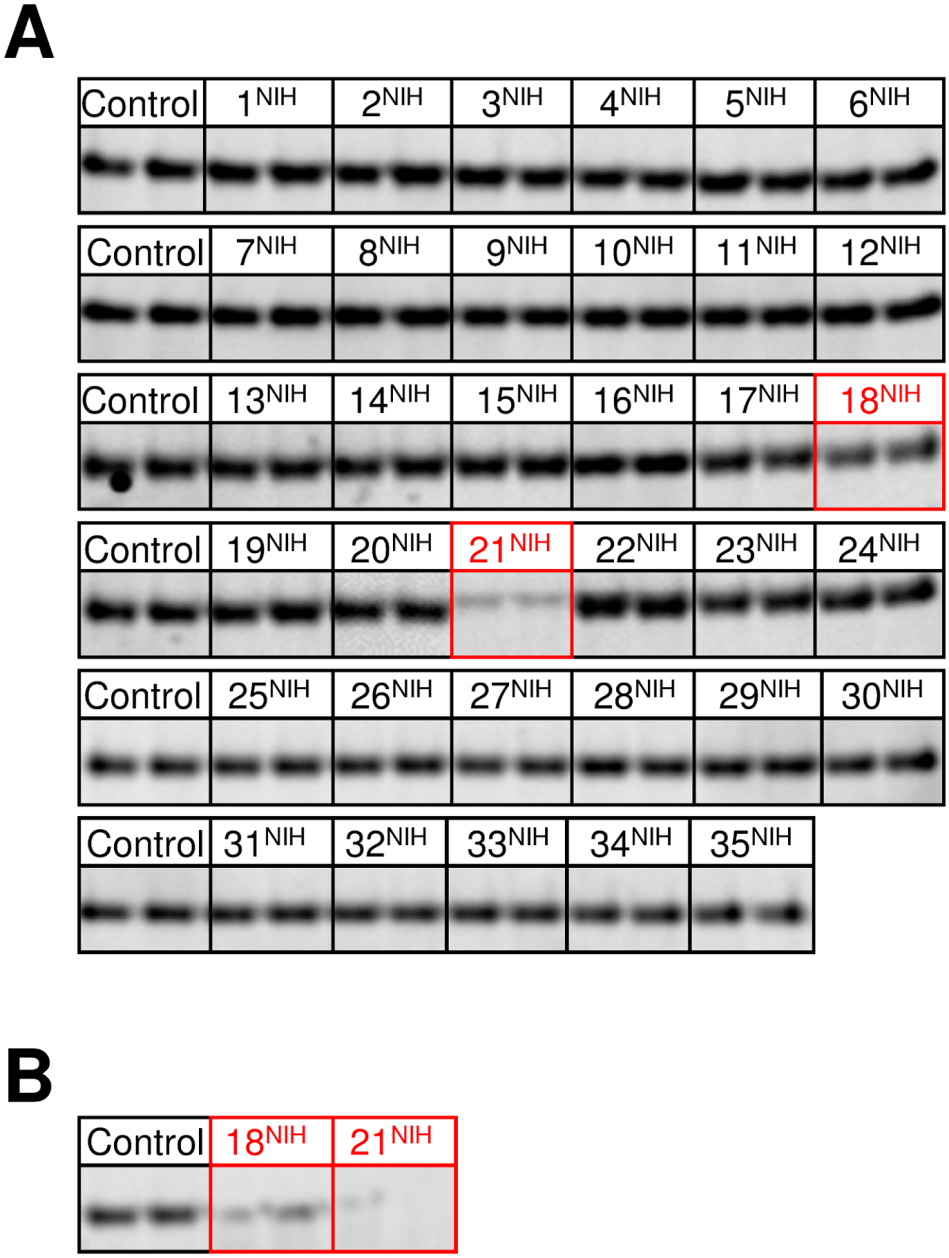
Effect of Hits from the BIPDeC HTS on *In Vitro* Tat Transport. (A) & (B) Tat transport assays as in (A) and (B) of Fig 7, respectively, using hit compounds from the BIPDeC HTS. Hit compound concentrations of 100 μM were used to maximize Tat transport inhibition, as the effects of a lower concentration (20 μM) were not very pronounced (data not shown). Compounds 18^NIH^ and 21^NIH^ survived the NADH-dependent assay (A) and were tested in the ATP-dependent assay (B). Both of these compounds survived the ATP-dependent assay. Surviving compounds at each stage are boxed in *red*.

Of the 35 compounds surviving the BIPDeC's HTS assay, bacterial growth assay and chemical evaluation, 2 compounds (S2H and S2I Fig) survived the NADH- and ATP-dependent Tat transport assays (Fig 9).

### Secondary Screening: Δψ Assay

Hit compounds surviving the *in vitro* Tat transport assay screens could be general Δψ inhibitors (ionophores), as both NADH and ATP generate the Δψ necessary for Tat transport. Since our current assay to measure Δψ requires ~20 times more test compound than the Tat transport assays, we reserved this assay for last. The presence of a Δψ was determined by measuring oxonol VI fluorescence [7]. For all 8 hit compounds that survived initial secondary screening, we tested their dose dependent effect on the Δψ, on NADH-dependent *in vitro* Tat transport efficiency, and on the fluorescence signal recovered from our primary HTS assay (live cell assay). An example of such an analysis is shown in S3 Fig. The *IC*_*50*_'s calculated from these data indicate that all surviving hit compounds, except compound 18^NIH^, collapse the Δψ (Table 1), and this explains their ability to inhibit Tat transport. Initially, compound 18^NIH^ (Broad Institute ID: BRD-A27765931-001-04-0) yielded promising results, as it did not collapse the Δψ across IMVs, and yet it did inhibit *in vitro* Tat transport during early trials (S4 Fig). However, these results could not be reproduced consistently. Out of 22 independent assays by three different people, compound 18^NIH^ inhibited Tat transport 12 times. The conflicting results seem more likely to arise from differences in IMV preparations rather than decomposition of or impurities in the samples of compound 18^NIH^.

**Table 1:** *IC*_*50*_'s for Hits Surviving the *In Vitro* Tat Transport Assays.

Compound ID	*IC*_*50*_ (μM) (Live Cell Assay)	*IC*_*50*_ (μM) (Tat Transport)	*IC*_*50*_ (μM) (Δψ)
1^JS^	6	3	1
51^JS^	20	12	8
111^JS^	31	0.7	0.1
181^JS^	20	1	0.4
202^JS^	5	11	3
140^JS^	14	2	1
18^NIH^	2	IC^a^	> 100^b^
21^NIH^	13	50	4

**Fig 10:**
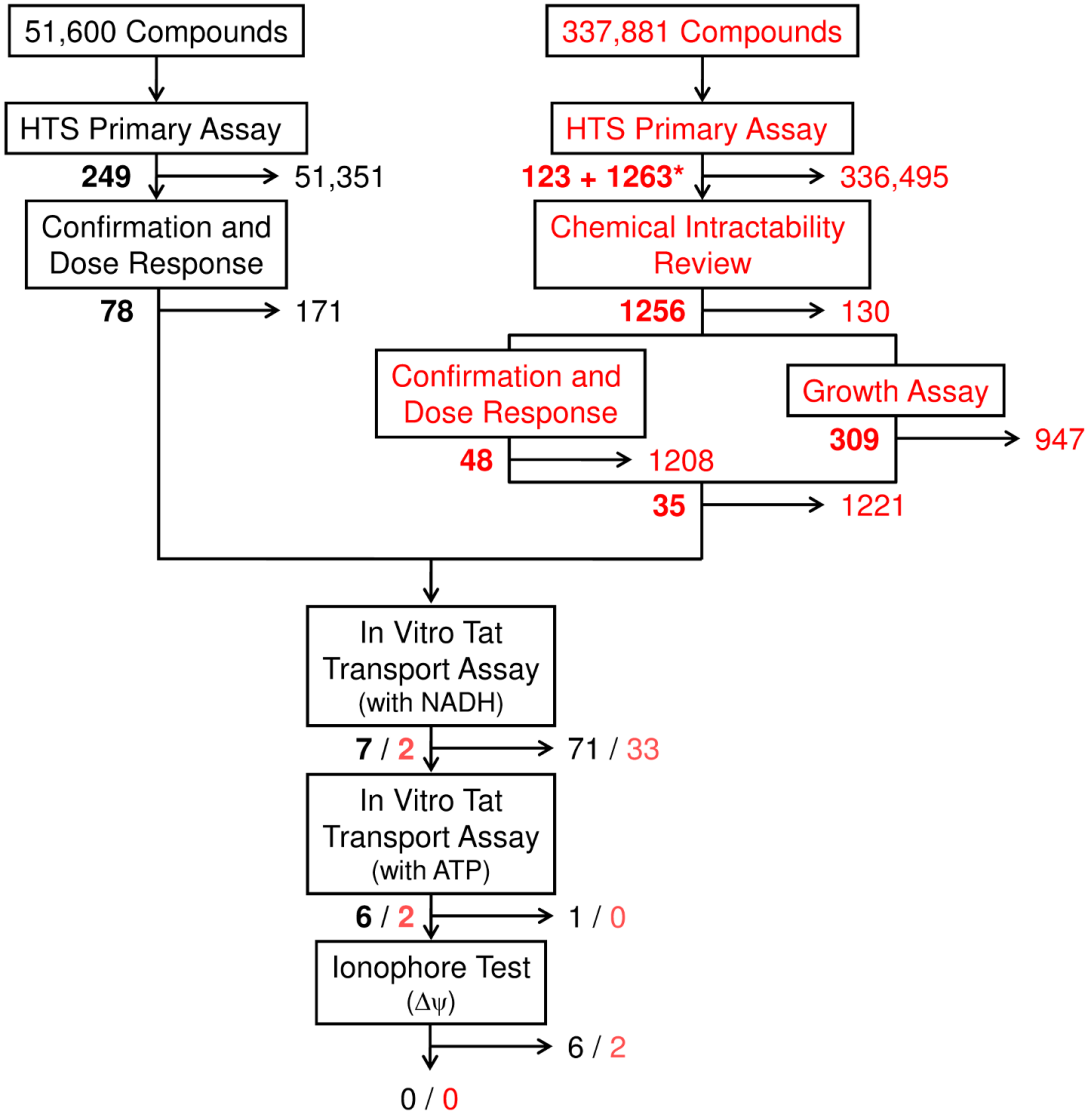
Summary of the HTS's and Hit Analysis by Follow-up Screens. Surviving and eliminated compounds from the local (*left*) and BIPDeC (*right*) HTS's are denoted by *black* and *red* numbers, respectively. The number of eliminated compounds at each step is shown to the right of the arrows and the number of surviving compounds are shown on the left in bold. Active (123) and inconclusive (1263*) compounds after the primary BIPDeC HTS were evaluated for chemical intractability and then retested in dose response and cell growth assays.

The *IC*_*50*_'s obtained for the mCherry fluorescence intensity during *in vivo* cell growth (column 2), for the efficiency of NADH-dependent *in vitro* Tat transport (column 3), and for the electrical field gradient (column 4) are tabulated for hit compounds that survived both *in vitro* Tat transport assays (see Fig 10). Sample results and assay details are provided in S3 Fig. Note that the membrane concentration in the *in vitro* transport assays (*A*_*280*_ = 5) was 10-fold higher than in the Δψ assays (*A*_*280*_ = 0.5), and hence, the *IC*_*50*_'s are higher in the former assays since the compounds must partition into the membrane bilayer. Transport assays require high IMV concentrations in order to observe sufficient transport on gels. Δψ assays require low IMV concentrations in order to more accurately detect the maximum Δψ before the dissolved oxygen is consumed [7].

^a^Inconclusive (IC) due to lack of reproducible inhibition of Tat transport (S4A Fig).

^b^Notably, 18^NIH^ increased the duration of the Δψ (S4B and S4C Fig).

### Effect of *Pseudomonas aeruginosa* Tat Inhibitors on *E*. *coli* Tat Transport

The two compounds *N*-phenylmaleimide and Bay 11–7082 were recently identified as inhibitors of the *Pseudomonas aeruginosa* Tat machinery as a result of high throughput screening [30]. We tested the effect of both compounds on transport by the *E*. *coli* Tat system. Both *N*-phenylmaleimide and Bay 11–7082 inhibited *E*. *coli* growth under Tat^++^ conditions (S5 Fig). Both compounds also inhibited synthesis of spTorA-mCherry-SsrA, though this appears to be a consequence of growth inhibition. Most importantly, neither compound affected *in vitro E*. *coli* Tat transport, and thus, we conclude that these compounds are not *E*. *coli* Tat transport inhibitors (S5 Fig).

Both *N*-phenylmaleimide and Bay 11–7082 chemically react with cysteines [40]. There is only one cysteine found in the *P*. *aeruginosa* Tat machinery, C208 of TatC. However, this residue is buried within the TatC structure [15], and thus, it seems unlikely that it can be modified by these reagents. Alternatively, if these reagents inhibit *P*. *aeruginosa* Tat transport by non-covalent binding interactions with Tat proteins, our data indicate that such interactions are not conserved between *E*. *coli* and *P*. *aeruginosa*.

## Discussion

A high throughput screening approach suitable for identifying small molecule inhibitors of the *E*. *coli* Tat protein transport system was developed. The strengths of the reported approach include: 1) an average Z'-factor > 0.7 (under the right screening conditions), which indicates a robust HTS assay that need not be run in duplicate [36]; 2) a live cell primary HTS, enabling early assessment of *in vivo* activity; 3) a hit rate ≤ ~0.5%, indicating selectivity; and 4) a series of *in vitro* follow-up screens/assays that enable direct evaluation of hit activity against the target system. Additional assays are available that would enable further *in vitro* characterization of compound binding properties and the site of action [7,41]. Unfortunately, despite screening ~380,000 compounds, no inhibitors that bind directly to Tat proteins were identified. Based on the *in vitro* follow-up assays, 7 hit compounds inhibit Tat transport by collapsing the Δψ, one compound (55^JS^) inhibits Δψ generation by NADH (possibly by inhibiting NADH dehydrogenase), and one compound (18^NIH^) yielded irreproducible results. In addition, the two compounds previously identified as *P*. *aeruginosa* Tat machinery inhibitors were shown not to be *E*. *coli* Tat inhibitors.

Since the proton motive force drives a wide range of crucial processes, *E*. *coli* are not expected to divide and multiply if they cannot maintain a membrane potential across the cytoplasmic membrane. How, then, did 7 hits survive the HTS and *in vivo* growth screen, only to be eliminated in follow-up assays as compounds that collapse the Δψ? Similarly, why do the ionophores nigericin and valinomycin inhibit Tat transport *in vitro* [7] but not *in vivo* (Fig 4A)? Efflux pumps, poor cell permeability of the cell envelope, or reduced efficacy due to binding interactions that reduce the compound's concentration in the lipid can certainly contribute to these results. However, if these are the only explanations, it is not immediately clear how compounds can reduce Tat transport (i.e., decrease total mCherry fluorescence) by disrupting the membrane potential and yet not affect cell growth. The answer may lie in the *magnitude* of the Δψ required to support Tat transport. We earlier reported that *in vitro E*. *coli* Tat transport requires a relatively large Δψ, though it is not necessary for the duration of the transport cycle [7]. Consequently, it is possible that *in vivo* Tat transport is substantially reduced when the Δψ is minimally decreased, and yet growth is largely unaffected since it is not as sensitive to the magnitude of the Δψ.

Compound 18^NIH^ (triclosan; PubChem CID 5564) has antibacterial and antifungal activities and is found in many commercial products, including soaps, toothpaste, hand and mouth washes, toys, bedding, and trash bags [42]. Surgical scrubbing with triclosan is effective against bacterial infections in a hospital setting [43]. Though triclosan has multiple cellular targets, the primary (high *K*_*D*_) target is the bacterial enzyme enoyl-acyl carrier protein reductase (ENR; gene *FabI*), an enzyme required for fatty acid synthesis [44]. Triclosan is bacteriostatic at low concentrations. At higher concentrations, it is bactericidal and it promotes tumors in liver [45,46]. We conclude that the inconsistent effects of triclosan on *in vitro* Tat transport arise from binding interactions with other target enzymes.

High throughput screening provides the opportunity to efficiently and cost-effectively test a large number of diverse small molecules such that at least a few lead compounds that inhibit the target pathway can be identified even when the “hit” rate is particularly low [47]. The identification of compounds that bind to transmembrane regions has been especially difficult [48–50]. Though TatA and TatB have extramembraneous cytoplasmic domains, these are relatively small and may not have stable secondary and tertiary structures [51]. TatC is predominantly transmembraneous, with relatively small extramembrane loops [15,18]. In addition, the signal peptide appears to bind deep within the membrane-spanning protein of the TatBC complex, rather than at the cytoplasmic surface [52,53]. Thus, the Tat machinery appears to have little hydrophilic surface area and it is unclear how much of this is important for binding interactions or conformational changes. These reasons may at least partially explain the difficulty with identifying inhibitors of the Tat machinery. Chemical libraries designed to target transmembrane regions may be better suited for identifying Tat machinery drug leads. Alternatively, the recent X-ray structure of TatC [15,18] suggests that identification of the signal peptide binding site is forthcoming, which would enable structure-based drug design.

## Materials and Methods

### Bacterial Strains, Plasmids and Growth Conditions

Plasmids and Tat proteins were as follows. Plasmids pTorA-GFP and pET25-SufI [7] encode spTorA-GFP-H6 and pre-SufI-H6, respectively, both of which contain C-terminal 6xHis tags. Plasmid pSufI-IAC [54] encodes a single-cysteine derivative of pre-SufI-H6, and was generated by mutations C17I and C295A and the addition of a cysteine immediately following the 6xHis tag (C497). Plasmids pTorA-mCherry-H6 and pTorA-mCherry-SsrA are derivatives of plasmid pTorA-GFP [7], and encode spTorA-mCherry-H6 and spTorA-mCherry-SsrA (S1 Fig), respectively, under the control of the arabinose inducible promoter of pBAD24 (ColE1 origin). These proteins consist of the TorA (a native Tat substrate) signal peptide (spTorA), mCherry (a fluorescent cargo protein) and either a 6xHis tag or SsrA-tag (which promotes protein degradation in the cytoplasm by the Clp protease system) [31]. Plasmid pTorA-GFP-SsrA encodes the protein spTorA-GFP-SsrA, which is identical to the TorA-GFP-SsrA protein reported earlier [31], except that the host vector is the same as for pTorA-mCherry-SsrA described above. Plasmid pTatABC-Duet1 was constructed by cloning the *Nco*I-*Sal*I insert from pTatABC [55] into pACYC-Duet1 (p15A origin). This plasmid encodes the *E*. *coli* TatA, TatB and TatC proteins under the control of T7 promoter, and can co-exist with plasmids pTorA-mCherry-H6, pTorA-mCherry-SsrA, or pTorA-GFP-SsrA. All protein coding regions were confirmed by DNA sequencing. The three different strain backgrounds used are: ΔTat (MC4100ΔTatABCDE); Tat^+^ [MC4100(DE3)]; and Tat^++^ [MC4100(DE3) with pTatABC-Duet1]. *E*. *coli* strains MC4100ΔTatABCDE and MC4100(DE3) were a gift of Tracy Palmer (University of Dundee), and have been described earlier [56,57].

*E*. *coli* strains MC4100(DE3) and JM109 [55,57,58] were used for the HTS and for cloning purposes, respectively. *E*. *coli* cultures were grown using Luria-Bertani (LB) media (Growcells, Irvine, CA 92618, USA) and were supplemented with ampicillin (Ap, 100 μg/mL; Sigma-Aldrich Corporation, MO, USA), chloramphenicol (Cm, 34 μg/mL) (USB Corporation, OH, USA), arabinose (Sigma-Aldrich Corporation, MO, USA), and IPTG (G-Biosciences, MO, USA) as required for growth and induction, unless otherwise indicated.

### Isolation of Arabinose-Resistant Strains

MC4100 is an *araD* mutant strain, and L-arabinose inhibits its growth. Suppressor mutations are readily obtained when this strain is grown with arabinose, and these typically yield strains generating variable levels of overproduced proteins [59]. Therefore, arabinose-resistant Tat^+^ and Tat^++^ strains were generated, as described below, to ensure more consistency in protein overproduction. An arabinose-resistant ΔTat strain was not generated.

*E*. *coli* strains MC4100(DE3) (pTatABC-Duet1, pTorA-mCherry-H6) and MC4100(DE3) (pTatABC-Duet1, pTorA-mCherry-SsrA) were grown in 20 mL LB media supplemented with Cm and Ap, and shaken (250 rpm) at 37°C until *A*_*500*_ ≈ 0.5. The cultures were then supplemented with 100 mM arabinose, incubated overnight (~15 hours) under the same conditions, and then serially diluted and plated on LB-agar supplemented with Cm and Ap. After incubating the plates overnight at 37°C, 6 colonies from each strain were tested for the production and periplasmic localization of spTorA-mCherry-H6 and spTorA-mCherry-SsrA by in situ gel-fluorescence using a phosphorimager Model FX. For each protein, all colonies exhibited similar production and periplasmic localization patterns (data not shown). This approach yielded strains that overproduce spTorA-mCherry-H6 or spTorA-mCherry-SsrA in the Tat^++^ background.

To obtain the Tat^+^ strain, a plasmid-free version of arabionose-resistant MC4100(DE3) (pTatABC-Duet1, pTorA-mCherry-SsrA) was obtained as follows. A 10 mL LB culture was grown overnight (37°C), and then repeatedly (5X) subcultured into fresh 10 mL LB media at a 1:100 ratio every 24 h. The final culture was serially diluted and plated on LB-agar plates and incubated overnight at 37°C. Colonies were replica-plated on LB-agar plates lacking antibiotics and plates supplemented with Ap or Cm, respectively. Six antibiotic-insensitive colonies were chosen, and confirmed to be arabinose-resistant by growth on plates with 100 mM arabinose. One of these was chosen as the Tat^+^ strain, which was transformed with pTorA-mCherry-SsrA to generate a strain that overproduces spTorA-mCherry-SsrA in the Tat^+^ background.

### Cell Fractionation

The cellular location of mCherry proteins was determined by cell fractionation. Cells were grown overnight in 2 mL LB broth supplemented with appropriate antibiotics at 37°C. These cultures were diluted 1:50 in 2 mL of fresh media, and growth was continued at 37°C with shaking (200 rpm) until *A*_*500*_ ≈ 1.0. Protein production was then induced by arabinose addition, and cultures were grown at 25°C for 8 h. Cells (1 mL, *A*_*500*_ = 1.0) were fractionated as described [60]. Both cytoplasmic and periplasmic fractions were suspended in a final volume of 100 μL in 1x sample buffer (10% glycerol, 2% SDS, 0.01% bromophenol blue, 1.25% β-mercaptoethanol, 60 mM Tris-Cl, pH 6.8), and a 10 μL fraction was analyzed by SDS-PAGE and detected by in situ gel-fluorescence using a phosphorimager Model FX (Bio-Rad Laboratories) [7], or by Western blot analysis.

### Imaging mCherry Localization

Imaging of cells expressing various mCherry precursors was performed similar to the approach described earlier for spTorA-GFP [60]. Strains were grown in 1 mL LB media supplemented with appropriate antibiotics at 37°C, 250 rpm for 6 h. mCherry Tat precursors were induced with 2 mM arabinose and cultures were incubated overnight (~15 h, 25°C, 250 rpm). Cells were harvested (4,000 *g* for 10 min), and incubated in fresh LB media (2 mL) with antibiotics but no arabinose for an additional 5 h. The undiluted culture (5 μL) was place on a glass coverslip, and covered with a 2 mm thick square of agarose gel (prepared in 17 mM NaCl, 10 mM Tris, pH 7.0) to immediately immobilize the cells. Cells were imaged with spinning disk confocal microscopy (Yokogawa) using 561 nm excitation on a Zeiss Axiovert 200M microscope equipped with a 1.46 NA 100X oil-immersion objective (Zeiss alpha PlanApochromat) and a 512x512 EMCCD camera (Andor) [61].

### Assays

Isolation of inverted membrane vesicles (IMVs), Western blot analyses and purification of pre-SufI-IAC were performed as described previously [7,62]. Western blots were performed using rabbit anti-mCherry antibodies (1:10,000 dilution) in the presence of 0.1% Triton X-100 and 0.1% Tween 20. The pre-SufI-IAC protein was labeled with Atto565 maleimide, and the resultant fluorescent protein, pre-SufI-IAC^Atto565^, was used as a substrate for the *in vitro* Tat transport assays (*A*_*280*_ = 5.0), as described previously [7,62]. The presence of a Δψ was determined by measuring oxonol VI fluorescence in presence of Tat^+^ IMVs (*A*_*280*_ = 0.5), as described previously [7], except that the translocation buffer lacked BSA. Steady-state fluorescence spectra and fluorescence intensities of whole cells producing mCherry proteins were determined with an SLM-8100 spectrofluorometer.

### HTS for 384-Well Plates

A single colony of *E*. *coli* strain MC4100(DE3) (pTatABC-DUET1, pTorA-mCherry-SsrA) was inoculated into a 100 mL LB culture supplemented with Ap and Cm and grown at 37°C with shaking (250 rpm) overnight. Cells were harvested by centrifugation at 4,000*g* for 10 min at room temperature (RT), suspended in 10 mL fresh LB broth supplemented with Ap and Cm, and the optical density (*A*_*500*_) was determined. Simultaneously, 700 mL LB supplemented with 100 mM arabinose, 2 mM IPTG, 100 μg/mL Ap and 68 μg/mL Cm was filter-sterilized (0.22 μm), and then divided into 200 mL (A) and 500 mL (B) portions. Sterile NaSCN (30 mM final; 1.5 mL of 4 M NaSCN) was added to portion A, and an equal ratio of sterile water was added to portion B (3.75 mL). Media and cells were transferred to the HTS facility on ice.

A CyBi-Well vario pipetting workstation (CyBio, Jena, Germany) was used to dispense library compounds and cultures. Library compounds (1 μL) solubilized in DMSO were added to 384-well black, clear-bottomed plates (Greiner Bio-one, Germany) in columns 3 to 22 (320 wells/plate). For controls, DMSO (1 μL) was added to each well in columns 1, 2, 22 and 23, to achieve DMSO uniformity across the plate. Both portions of LB media were inoculated with *E*. *coli* MC4100(DE3) (pTatABC-DUET1, pTorA-mCherry-SsrA) from the concentrated stock to a final *A*_*500*_ = 0.15. The media with 30 mM NaSCN (positive control, or simulated hit) was dispensed into each well of columns 1 and 24 (49 μL each). An equivalent volume of media without NaSCN was dispensed into each well of columns 2 to 23. Columns 2 and 23 lacked NaSCN and test compounds and served as negative controls. The first 12,800 compounds were screened at 5 μM (plates 1–40), and the remaining 38,800 compounds were screened at 20 μM (plates 41–162).

The plates containing cells and dispersed test compounds were grown with shaking (200 rpm) at 25°C for 8 h. Plates were individually mounted, not stacked, on a hollow plastic platform, allowing adequate air circulation. Following the 8 h incubation, plates were transferred to 4°C for 16 h. Each plate was covered with an empty plate to prevent condensation on the lid. The overnight incubation was found to improve the signal-to-background ratio. Following the incubation, plates were read using a BMG Polarstar Omega plate reader (BMG Labtech, Ortenberg, Germany) to determine optical densities (*A*_500_) and mCherry fluorescence intensities (EX = 584 nm, EM = 620 nm).

### HTS for 1536-Well Plates

This assay was performed at the Broad Institute Probe Development Center (BIPDeC; Cambridge, MA), a comprehensive screening center and member of the Molecular Libraries Probe Production Centers Network (MLPCN). The original 384-well assay was transferred to the BIPDeC, where the assay was miniaturized and optimized to run in 1536-well plates. Briefly, an *E*. *coli* MC4100(DE3) (pTatABC-Duet1, pTorA-mCherry-SsrA) culture was grown overnight in liquid LB broth supplemented with Ap and Cm. The culture was harvested, suspended in 10 mL fresh media, and placed on ice. The optical density of the culture was determined, and then diluted to *A*_*500*_ = 0.15 with ice cold LB broth supplemented with 100 mM arabinose, 2 mM IPTG, 100 μg/mL Ap and 68 μg/mL Cm. Black, solid bottom, 1536-well assay plates (Aurora (Brooks), Chelmsford, MA) were previously prepared with compounds in DMSO (7.5 nL) by acoustic transfer (Echo555, LabCyte, Sunnyvale, CA). Positive (LB broth supplemented with 30 mM NaSCN) and negative (LB broth supplemented with an equivalent volume of water instead of NaSCN) controls were added (100 nL) to 256 designated control wells. Assay plates were loaded into an integrated high throughput screening system (HighRes Biosolutions, Woburn, MA), which performed all the assay steps. The assay was initiated by the addition of 7.5 μL of the induced culture, and then incubated for 8 h at 30°C with shaking at 200 rpm. Plates were removed from the incubator, 2.5 μL of 5% glucose was added to each well, and the plates were returned to the incubator for 4 h. Plates were then incubated at 4°C overnight. At the completion of the assay, the plates were read on an Envision (PerkinElmer, Waltham, MA) plate reader to determine the fluorescence intensity. The data were analyzed using Genedata Assay Analyzer (Lexington, MA), and submitted to PubChem. Following analysis, compounds from the primary screen were ordered and retested at dose response. Compounds were spotted on assay plates as previously described, at 8 different concentrations ranging from 40 μM to 0.3 μM, in 2-fold dilutions. These same compounds were also tested in a counter assay designed to identify non-specific bacterial growth inhibitors, which produce false positive results in the primary assay. *E*. *coli* MC4100(DE3) (pTatABC-Duet1, pTorA-mCherry-SsrA) was cultured and harvested as done for the primary assay, and then inoculated into fresh LB supplemented only with Ap and Cm. Compounds were spotted in dose response format on white, solid-bottomed assay plates (Aurora (Brooks), Chelmsford, MA). Kanamycin (40 μg/mL) was added to 144 positive control wells. Cultures were incubated with compounds for 8 h, and then assessed for viability with Bac-TiterGlo (Promega, Madison, WI). Luminescence was detected with an Envision plate reader. Data from the dose response experiments were analyzed using Genedata Assay Analyzer and Condeseo, and submitted to PubChem.

### Characteristics of Chemical Libraries

The local library was constructed to ensure diversity among the compounds, guaranteeing that no pair of compounds had a Tanimoto similarity score [63] greater than 0.7. This approach minimizes the bias of many existing libraries, which often contain many variations of common scaffolds. This diversity library was designed by combining structures from catalogs of small molecules from multiple commercial vendors totaling over 3 million compounds, and selecting a diverse and representative subset using a clustering algorithm. The compounds were first filtered by Lipinski’s rules to remove non-drug-like compounds, and then compounds with reactive chemotypes or those predicted to be promiscuous aggregators were removed. At the time of the primary HTS assay, the local library consisted of 51,600 compounds.

The MLSMR collection of compounds is described at: http://mli.nih.gov/mli/compound-repository/mlsmr-compounds/. At the time of our primary screening, the MLSMR collection of compounds consisted of 337,881 compounds.

## Supporting Information

S1 Fig Restriction Map and Amino Acid Sequence of spTorA-mCherry-SsrA. The amino acid sequence of spTorA, mCherry and the SsrA tag are highlighted in *yellow*, *red* and *green*, respectively. The linker sequence between spTorA and mCherry is highlighted in *blue*. The parent plasmid was pTorA-GFP [7]. For pTorA-mCherry-H6, the SsrA-tag was replaced with a 6xHis-tag. For pTorA-GFP-SsrA, the mCherry protein was replaced with GFP.
(TIFF)

S2 Fig Chemical Structures of Hit Compounds that Survived the *In Vitro* NADH-Dependent Tat Transport Assays. (A) [(2-bromophenyl)hydrazono]malononitrile (1^JS^; PubChem CID 23273954), (B) 1-chloro-2,4-dinitronaphthalene (51^JS^; PubChem CID 16987), (C) N-(4,7-dioxo-2,1,3-benzoxadiazol-5-yl)acetamide (55^JS^; PubChem CID 610143), (D) 1-[(2,3,5,6-tetrafluoropyridin-4-yl)amino]-3-[(3-trifluoromethyl)phenyl]thiourea (111^JS^; PubChem CID 1825999), (E) 1-(5-nitro-1,3-thiazol-2-yl)-3-[3-(trifluoromethyl)phenyl]urea (181^JS^; PubChem CID 4416121), (F) 4-nitro-2-thiophen-2-ylphenol (202^JS^; PubChem CID 7131469), (G) 5-[3-(trifluoromethyl)phenyl]isoquinoline (140^JS^), (H) 5-Chloro-2-(2,4-dichlorophenoxy)phenol (18^NIH^; triclosan; PubChem CID 5564), and (I) 5-[(3,5-dichlorophenyl)methyl]-4,6-dihydrotriazolo[1,5-a][1,4]benzodiazepine-6-carbonitrile. (NIH^21^; PubChem CID 44825859).
(TIFF)

S3 Fig *IC*_*50*_'s for Hit Compound 181^JS^. These data illustrate how the *IC*_*50*_'s summarized in Table 1 were obtained. (A) The total cellular mCherry fluorescence (live cell assay; *n* = 1), (B) the *in vitro* NADH-dependent Tat transport efficiency of pre-SufI (*n* = 1), and (C) the maximum transmembrane electrical potential gradient (*n* = 1) were measured for a range of concentrations of compound 181^JS^ (S2E Fig). (D) SDS-PAGE gel showing in-gel fluorescence data used in (B). For (A), cells expressing spTorA-mCherry-SsrA were grown under Tat^++^ conditions in a 384-well plate in the presence of 0, 0.08, 0.15, 0.3, 0.63, 1.25, 2.5, 5, 10, 20, or 40 μM compound 181^JS^, as described for the HTS assay (see Methods). For (B) and (C), IMVs were incubated with the same concentrations of compound 181^JS^ prior to initiation of Tat transport and Δψ generation by the addition of NADH, as described previously [7]. The *red* curves are best-fits using *y* = *d* + (100-*d*)/(1+(*x*/*IC*_*50*_)^*b*^), where *b* is a slope factor and *d* is the high concentration asymptote [64].
(TIFF)

S4 Fig Characterization of Compound 18^NIH^. (A) Effect of compound 18^NIH^ (S2H Fig) on *in vitro* Tat transport of pre-SufI using NADH (4 mM) to generate a Δψ. Out of 22 independent assays by three different people, compound 18^NIH^ inhibited Tat transport 12 times. Shown here are 4 representative assays, the top 2 of which show inhibited transport. For calibration of transport efficiency, 20 and 40% of the pre-SufI added to the reaction is shown in the leftmost lanes (Std). A minus NADH control (-) is also shown. (B & C) Effect of compound 18^NIH^ on the magnitude and duration of the Δψ across the IMV membrane. The top panel (B) demonstrates that compound 18^NIH^ affected both the magnitude and the duration of the Δψ for the assayed IMV preparation. The bottom panel (C) summarizes the effect of compound 18^NIH^ on the duration of the Δψ, the magnitude of the Δψ and *in vitro* Tat transport (*n* = 1 for the Δψ values and *n* = 3 for *in vitro* Tat transport using two IMV preparations). These data suggest that when compound 18^NIH^ does inhibit Tat transport, it does NOT do so by collapsing the Δψ. However, since compound 18^NIH^ does NOT ALWAYS inhibit Tat transport (see A), direct binding to (and inhibition of) the Tat machinery does not appear to be the dominating interaction of the compound.
(TIFF)

S5 Fig Effect of *P*. *aeruginosa* Tat Inhibitors on *E*. *coli* Cell Growth and Tat Transport. The concentration-dependent effects of Bay 11–7082 (A) and *N*-phenylmaleimide (B) on bacterial growth *(black*) and *in vivo* Tat transport of spTorA-mCherry-SsrA *(red)* was determined under Tat^++^ conditions (*top*). An overnight culture of MC4100(DE3) (pTorA-mCherry-SsrA, pTatABC-Duet1) was diluted 1:50 in 10 mL fresh LB broth with appropriate antibiotics and dispensed (2 mL) into 5 tubes, and growth was continued at 37°C with shaking at 200 rpm until *A*_*500*_ ≈ 0.5. The indicated concentrations of the two compounds were added, and the expression of spTorA-mCherry-SsrA and TatABC were induced. Cultures were grown for another 8 h at 25°C. Cell growth was assayed by *A*_*500*_, and cells (1 mL, *A*_*500*_ = 1.0) were fractionated into cytoplasmic (C) and periplasmic (P) fractions as described [60]. Transport of spTorA-mCherry-SsrA was assayed by in-gel fluorescence of mCherry (*upper gels*) and Western blot analyses (*middle gels*). The quantified *in vivo* transport of spTorA-mCherry-SsrA is an average of the in-gel mCherry fluorescence and the Western blot analysis. The mature mCherry-SsrA in the periplasmic fraction of cells grown in the absence of a putative inhibitor was set to 100%. *In vitro* Tat transport assays in the presence of 2.5 mM DTT (*bottom gels*) were performed with 90 nM pre-SufI and reactions were initiated with NADH [7]. Control assays performed in the absence of NADH are identified (-). In the left set of experiments (I), the putative inhibitors were treated with DTT prior to addition of IMVs and pre-SufI. In the right set of experiments (II), IMVs were pre-incubated with the putative inhibitors prior to addition of pre-SufI and DTT. Transport assays performed with and without DTT (*bottom*; IMVs pre-incubated with compounds for 5 min prior to SufI and NADH addition) demonstrate that both DTT reacted and unreacted forms of the two compounds do not inhibit *E*. *coli* Tat transport.
(TIFF)
