# Supplementary figures and images for: High Throughput Screen for Escherichia coli Twin Arginine Translocation (Tat) Inhibitors
Source: PLoS One. 2016 Feb 22;11(2):e0149659. doi: 10.1371/journal.pone.0149659 (PMC4764201; doi:10.1371/journal.pone.0149659)

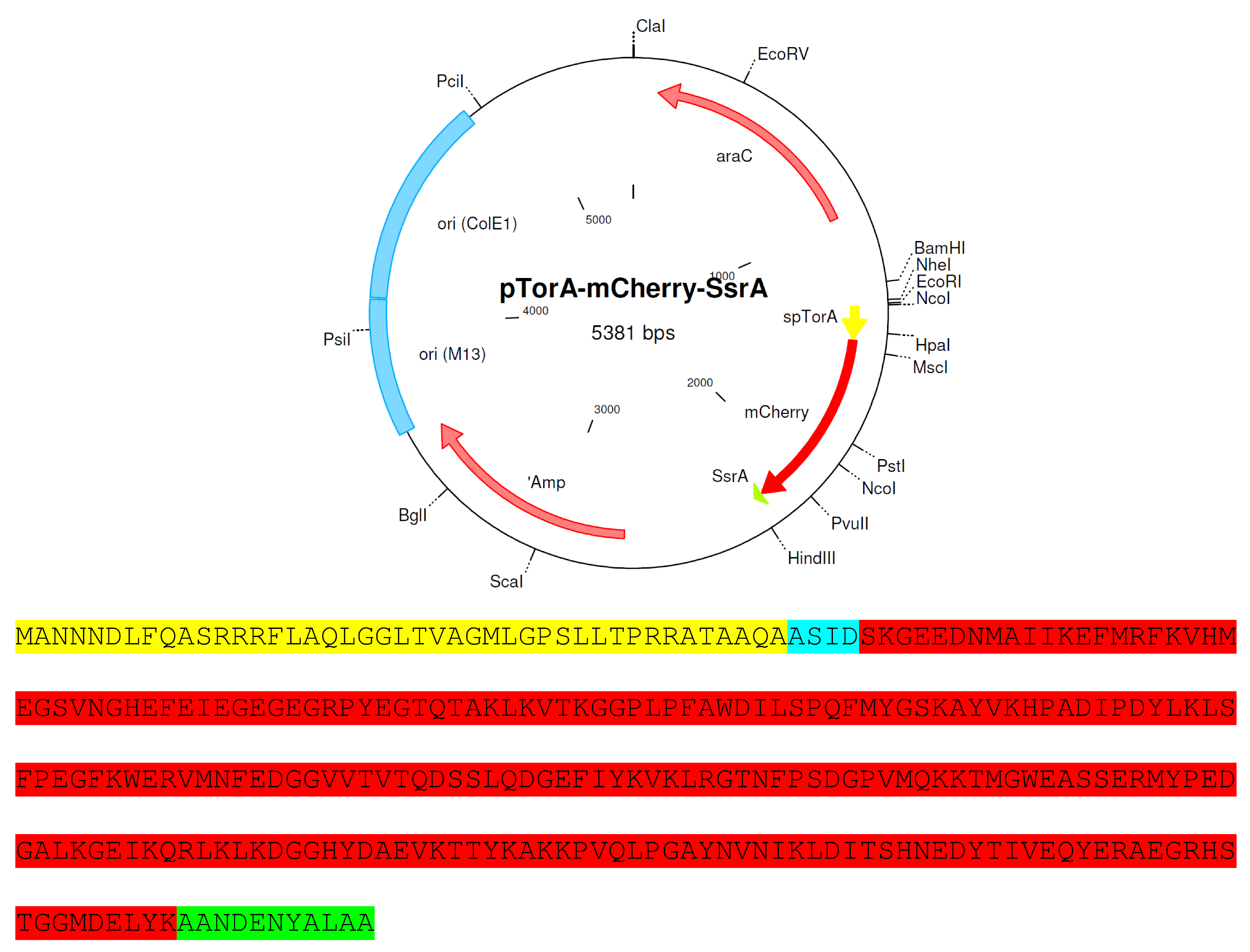

Supplement: S1 Fig — The amino acid sequence of spTorA, mCherry and the SsrA tag are highlighted in yellow, red and green, respectively. The linker sequence between spTorA and mCherry is highlighted in blue. The parent plasmid was pTorA-GFP [7]. For pTorA-mCherry-H6, the SsrA-tag was replaced with a 6xHis-tag. For pTorA-GFP-SsrA, the mCherry protein was replaced with GFP. (TIFF) [file pone.0149659.s001.tiff]

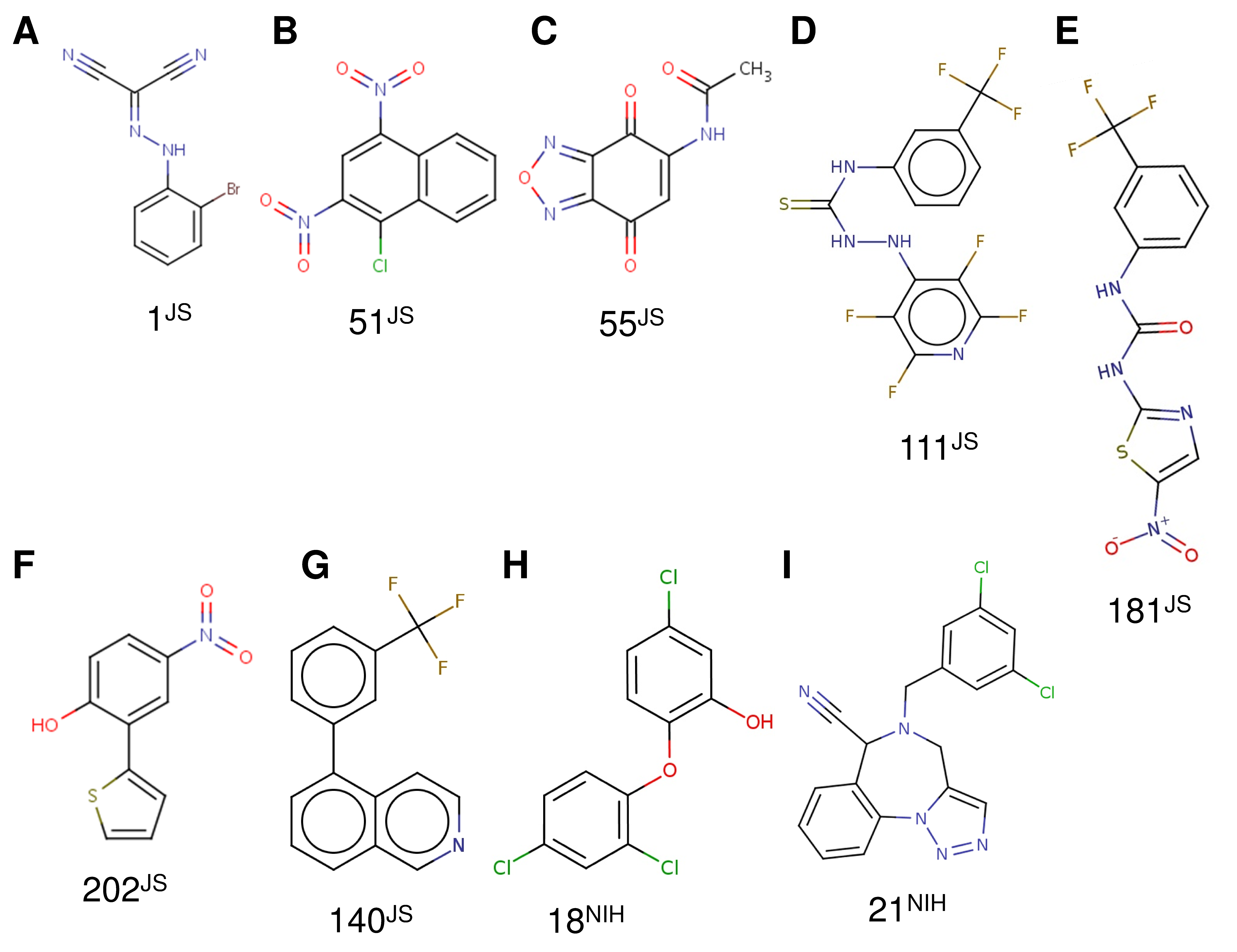

Supplement: S2 Fig — (A) [(2-bromophenyl)hydrazono]malononitrile (1JS; PubChem CID 23273954), (B) 1-chloro-2,4-dinitronaphthalene (51JS; PubChem CID 16987), (C) N-(4,7-dioxo-2,1,3-benzoxadiazol-5-yl)acetamide (55JS; PubChem CID 610143), (D) 1-[(2,3,5,6-tetrafluoropyridin-4-yl)amino]-3-[(3-trifluoromethyl)phenyl]thiourea (111JS; PubChem CID 1825999), (E) 1-(5-nitro-1,3-thiazol-2-yl)-3-[3-(trifluoromethyl)phenyl]urea (181JS; PubChem CID 4416121), (F) 4-nitro-2-thiophen-2-ylphenol (202JS; PubChem CID 7131469), (G) 5-[3-(trifluoromethyl)phenyl]isoquinoline (140JS), (H) 5-Chloro-2-(2,4-dichlorophenoxy)phenol (18NIH; triclosan; PubChem CID 5564), and (I) 5-[(3,5-dichlorophenyl)methyl]-4,6-dihydrotriazolo[1,5-a][1,4]benzodiazepine-6-carbonitrile. (NIH21; PubChem CID 44825859). (TIFF) [file pone.0149659.s002.tiff]

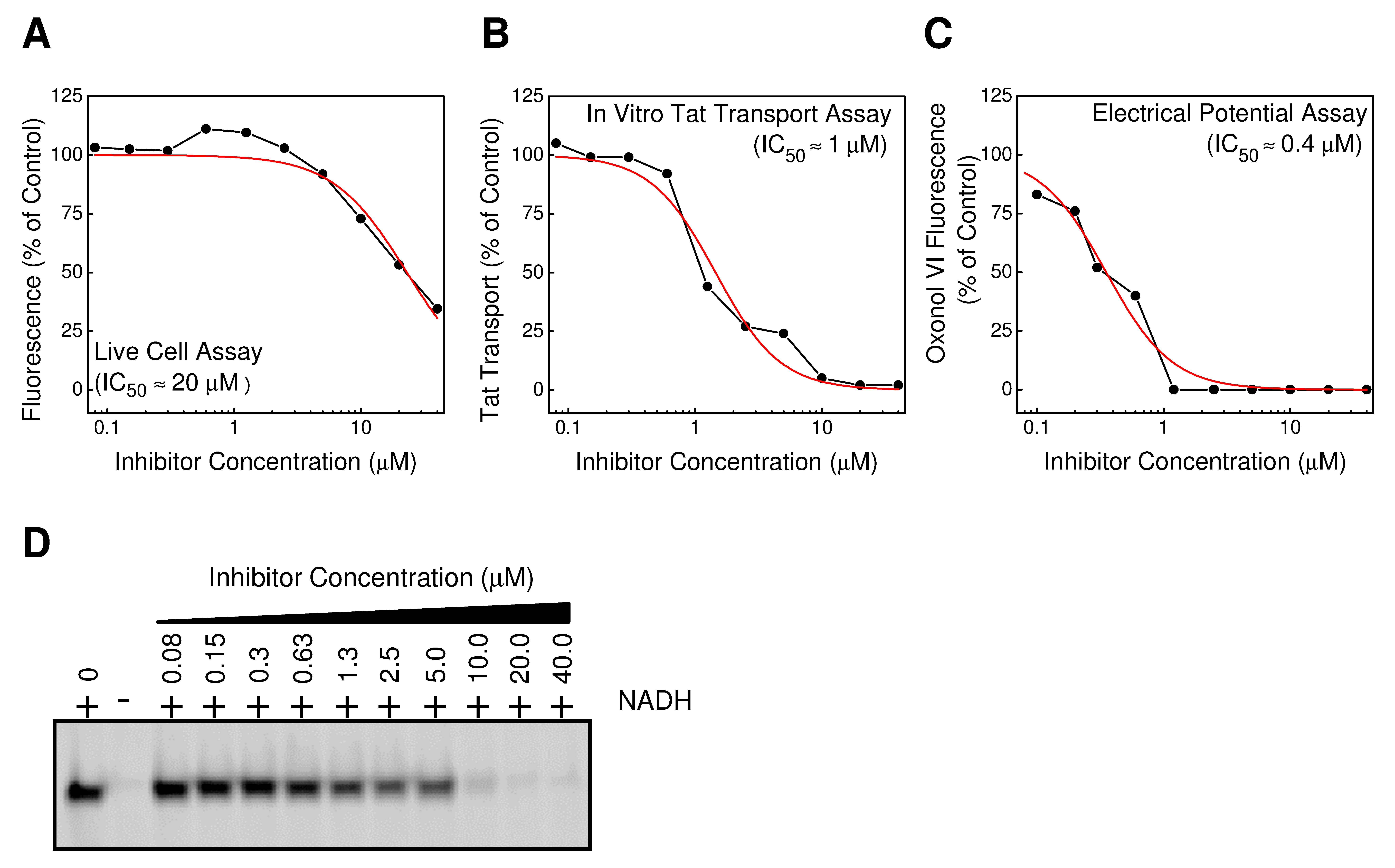

Supplement: S3 Fig — These data illustrate how the IC50's summarized in Table 1 were obtained. (A) The total cellular mCherry fluorescence (live cell assay; n = 1), (B) the in vitro NADH-dependent Tat transport efficiency of pre-SufI (n = 1), and (C) the maximum transmembrane electrical potential gradient (n = 1) were measured for a range of concentrations of compound 181JS (S2E Fig). (D) SDS-PAGE gel showing in-gel fluorescence data used in (B). For (A), cells expressing spTorA-mCherry-SsrA were grown under Tat++ conditions in a 384-well plate in the presence of 0, 0.08, 0.15, 0.3, 0.63, 1.25, 2.5, 5, 10, 20, or 40 μM compound 181JS, as described for the HTS assay (see Methods). For (B) and (C), IMVs were incubated with the same concentrations of compound 181JS prior to initiation of Tat transport and Δψ generation by the addition of NADH, as described previously [7]. The red curves are best-fits using y = d + (100-d)/(1+(x/IC50)b), where b is a slope factor and d is the high concentration asymptote [64]. (TIFF) [file pone.0149659.s003.tiff]

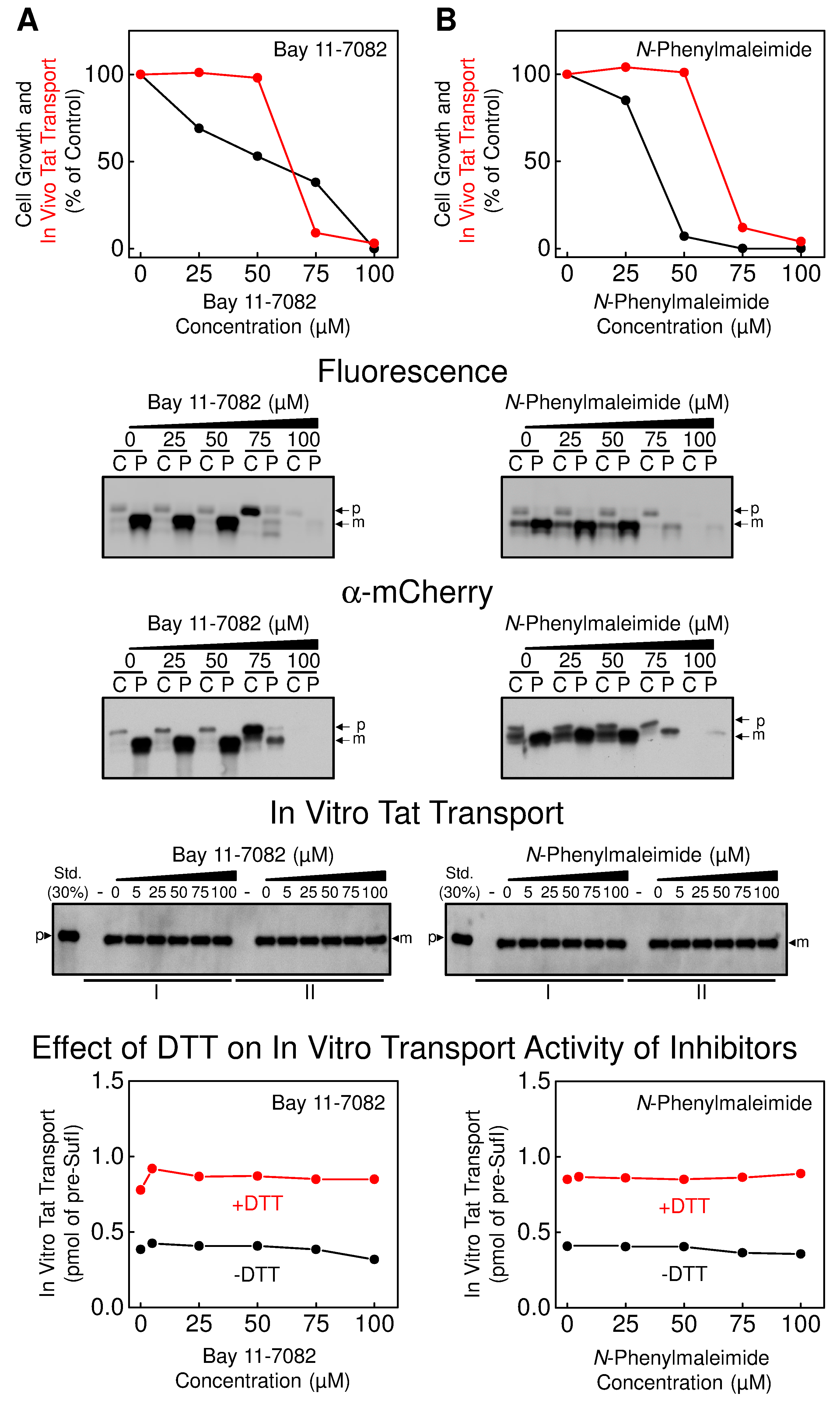

Supplement: S5 Fig — The concentration-dependent effects of Bay 11–7082 (A) and N-phenylmaleimide (B) on bacterial growth (black) and in vivo Tat transport of spTorA-mCherry-SsrA (red) was determined under Tat++ conditions (top). An overnight culture of MC4100(DE3) (pTorA-mCherry-SsrA, pTatABC-Duet1) was diluted 1:50 in 10 mL fresh LB broth with appropriate antibiotics and dispensed (2 mL) into 5 tubes, and growth was continued at 37°C with shaking at 200 rpm until A500 ≈ 0.5. The indicated concentrations of the two compounds were added, and the expression of spTorA-mCherry-SsrA and TatABC were induced. Cultures were grown for another 8 h at 25°C. Cell growth was assayed by A500, and cells (1 mL, A500 = 1.0) were fractionated into cytoplasmic (C) and periplasmic (P) fractions as described [60]. Transport of spTorA-mCherry-SsrA was assayed by in-gel fluorescence of mCherry (upper gels) and Western blot analyses (middle gels). The quantified in vivo transport of spTorA-mCherry-SsrA is an average of the in-gel mCherry fluorescence and the Western blot analysis. The mature mCherry-SsrA in the periplasmic fraction of cells grown in the absence of a putative inhibitor was set to 100%. In vitro Tat transport assays in the presence of 2.5 mM DTT (bottom gels) were performed with 90 nM pre-SufI and reactions were initiated with NADH [7]. Control assays performed in the absence of NADH are identified (-). In the left set of experiments (I), the putative inhibitors were treated with DTT prior to addition of IMVs and pre-SufI. In the right set of experiments (II), IMVs were pre-incubated with the putative inhibitors prior to addition of pre-SufI and DTT. Transport assays performed with and without DTT (bottom; IMVs pre-incubated with compounds for 5 min prior to SufI and NADH addition) demonstrate that both DTT reacted and unreacted forms of the two compounds do not inhibit E. coli Tat transport. (TIFF) [file pone.0149659.s005.tiff]
